# Supplementary material for: New Hybrid Phenalenone Dimer, Highly Conjugated Dihydroxylated C28 Steroid and Azaphilone from the Culture Extract of a Marine Sponge-Associated Fungus, Talaromyces pinophilus KUFA 1767
Source: Mar Drugs. 2023 Mar 21;21(3):194. doi: 10.3390/md21030194 (PMC10051590; doi:10.3390/md21030194)
Supplement: Supplementary file 1 [file marinedrugs-21-00194-s001.zip › marinedrugs-2277486-supplementary.pdf]

Supplementary Materials

New Hybrid Phenalenone Dimer, Highly Conjugated Dihydroxylated C_{28} Steroid and Azaphilone from the Culture Extract of a Marine Sponge-Associated Fungus, *Talaromyces pinophilus* KUFA 1767

Fátima P. Machado[1,2], Inês C. Rodrigues[1], Aikaterini Georgopolou[3], Luís Gales[1,4], José A. Pereira[1, 2], Paulo M. Costa[1, 2], Sharad Mistry[5], Salar Hafez Ghoran[6], Artur M. S. Silva[7], Tida Dethoup[8*], Emilia Sousa[2,9*], Anake Kijjoa[1, 2*]

[1] ICBAS-Instituto de Ciências Biomédicas Abel Salazar, Rua de Jorge Viterbo Ferreira, 228, 4050-313 Porto, Portugal
[2] Interdisciplinary Centre of Marine and Environmental Research (CIIMAR), Terminal de Cruzeiros do Porto de Leixões, Av. General Norton de Matos s/n, 4450-208, Matosinhos, Portugal

[3] Department of Pharmacy, National and Kapodistrian University of Athens, 15771 Athens, Greece

[4] Instituto de Biologia Molecular e Celular (i3S-IBMC), Universidade do Porto, Rua de Jorge Viterbo Ferreira, 228, 4050-313 Porto, Portugal
[5] Department of Chemistry, University of Leicester, University Road, Leicester LE 7 RH, UK
[6] HEJ Research Institute of Chemistry, International Center for Chemical and Biological Sciences, University of Karachi, Karachi 75270, Pakistan
[7] Departamento de Química & QOPNA, Universidade de Aveiro, 3810-193 Aveiro, Portugal
[8] Department of Plant Pathology, Faculty of Agriculture, Kasetsart University, Bangkok 10240, Thailand
[9] Laboratório de Química Orgânica e Farmacêutica, Departamento de Ciências Químicas, Faculdade de Farmácia, Universidade do Porto and CIIMAR, Rua de Jorge Viterbo Ferreira 228, 4050-313 Porto, Portugal
*Correspondence: ankijjoa@icbas.up.pt (A.K.); Tel. +351-962712474; Fax: +351-22-206-2232; esousa@ff.up.pt (E. S); Tel. +351-220428689; agrtdd@ku.ac.th (T.D.); Tel:+66-25791026.

Figure S1. ^{1}H NMR spectrum of **1** ($DMSO_{d6}$, 300 MHz).

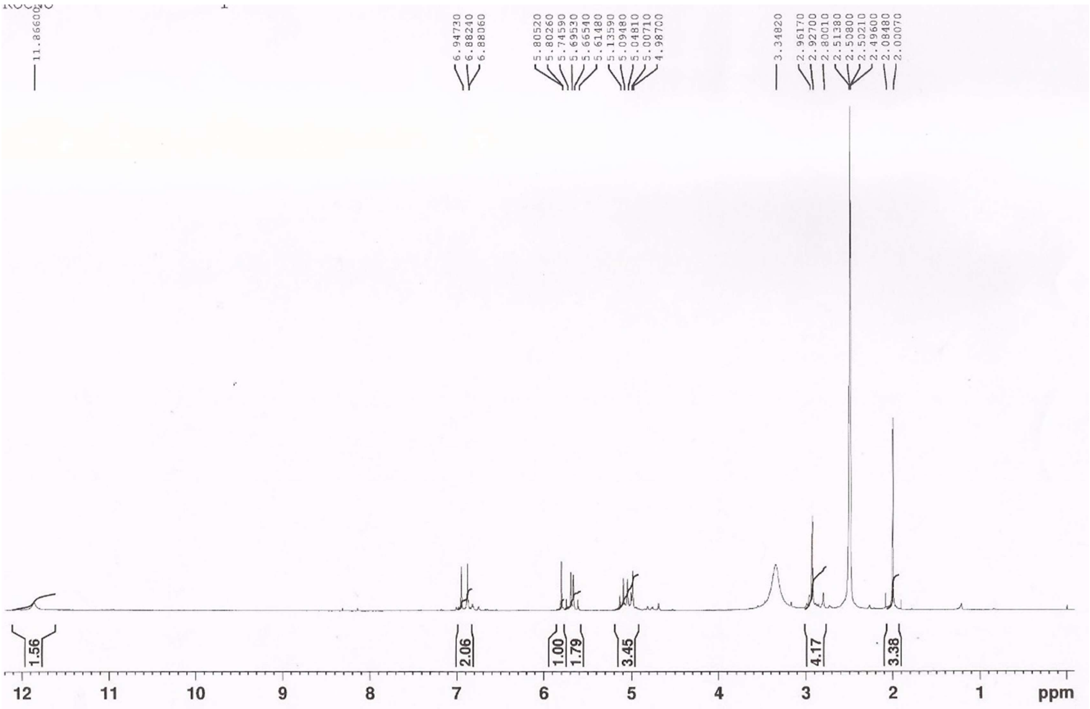

Figure S2. ^{13}C NMR spectrum of **1** ($DMSO_{d6}$, 75 MHz).

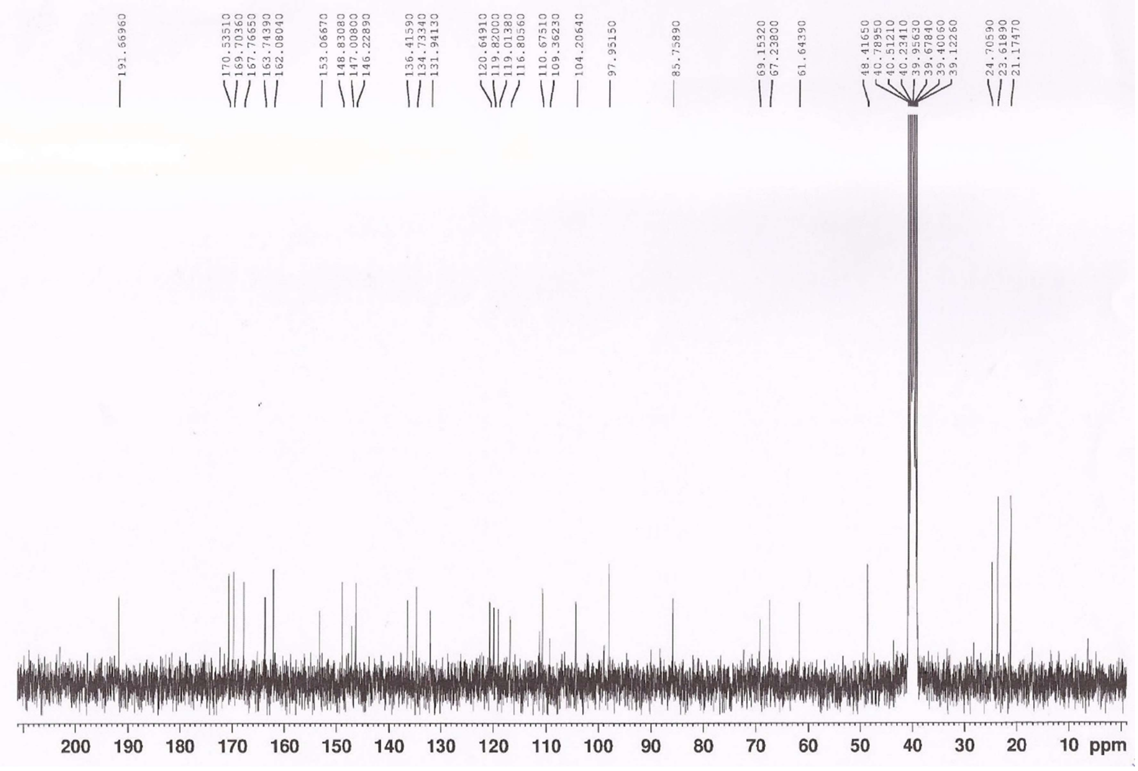

Figure S3. ^{1}H NMR spectrum of **2** (DMSO$_{d6}$, 300 MHz).

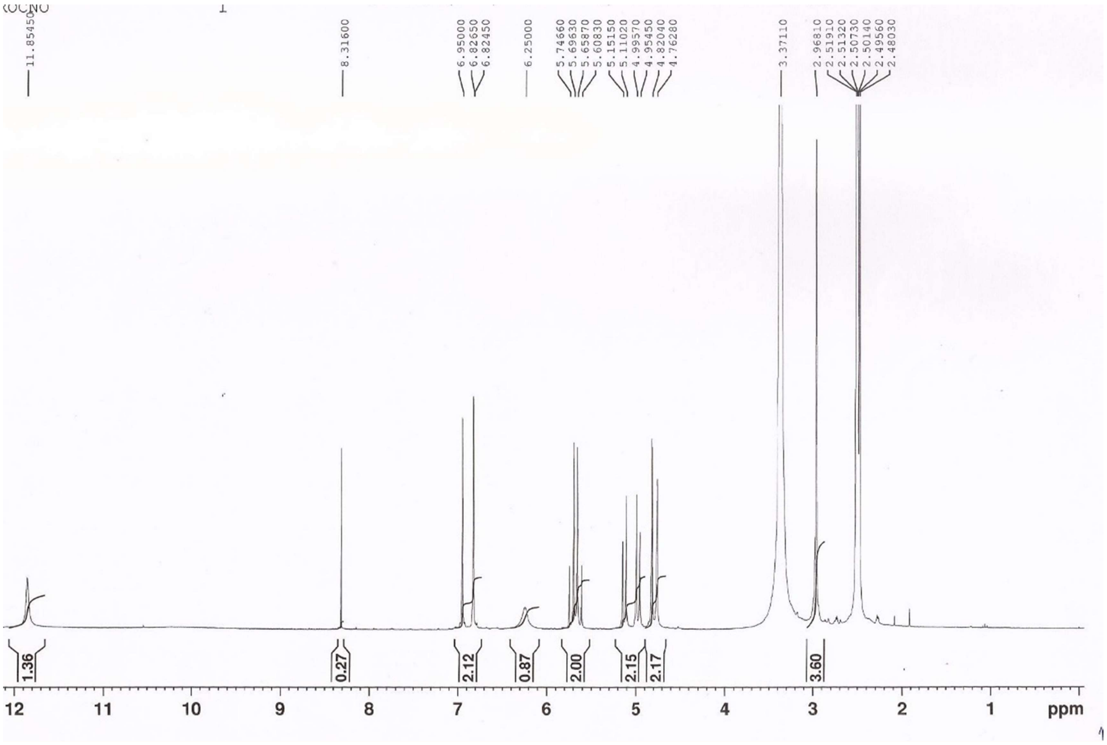

Figure S4. ^{13}C NMR spectrum of **2** (DMSO$_{d6}$, 75 MHz).

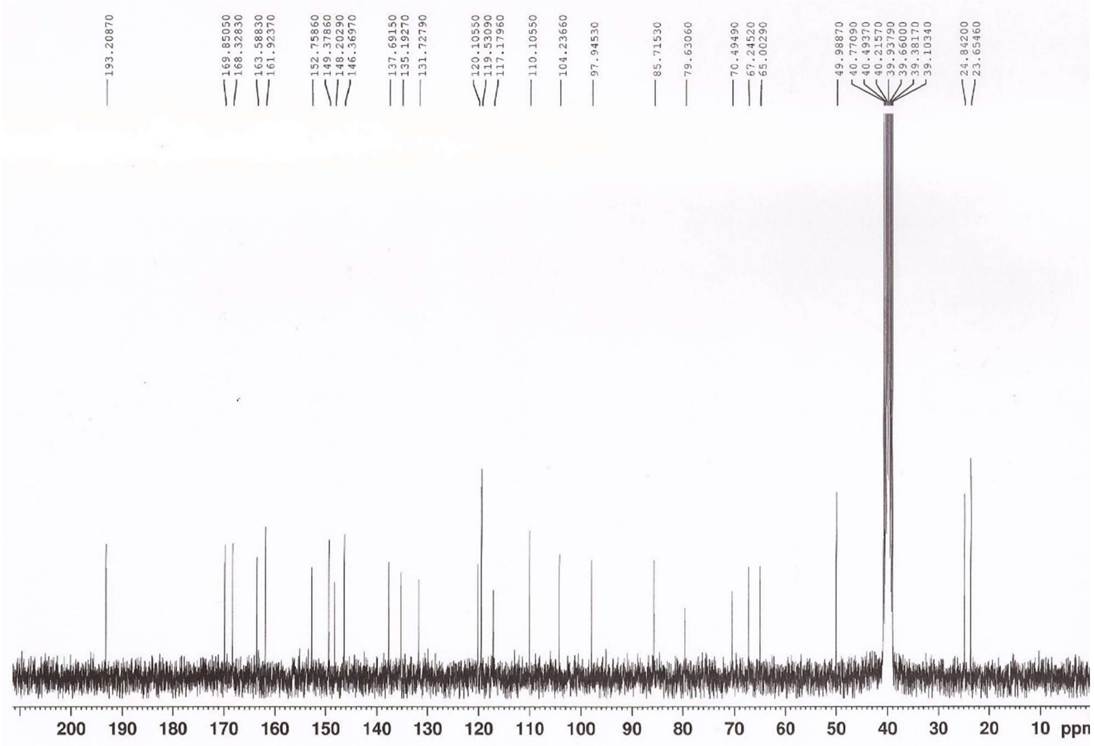

Figure 5 ROESY spectrum of **2** ($DMSO_{d6}$, 300 MHz).

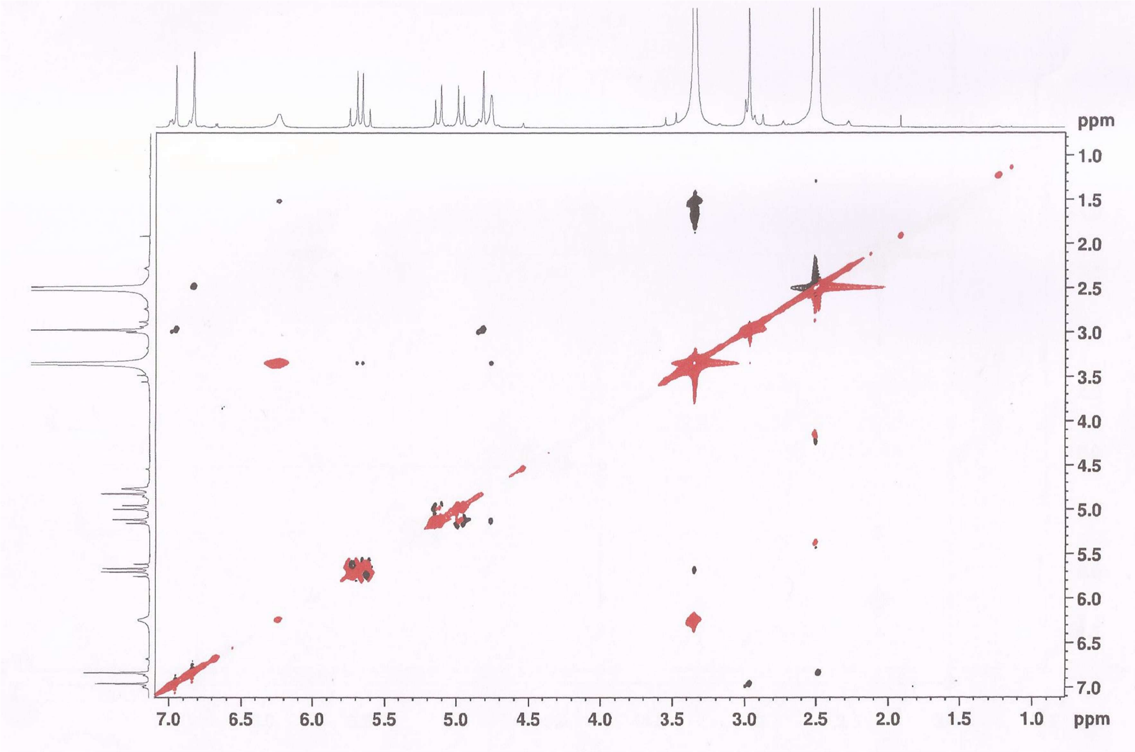

Figure S6. ^{1}H NMR spectrum of **3** ($DMSO_{d6}$, 300 MHz).

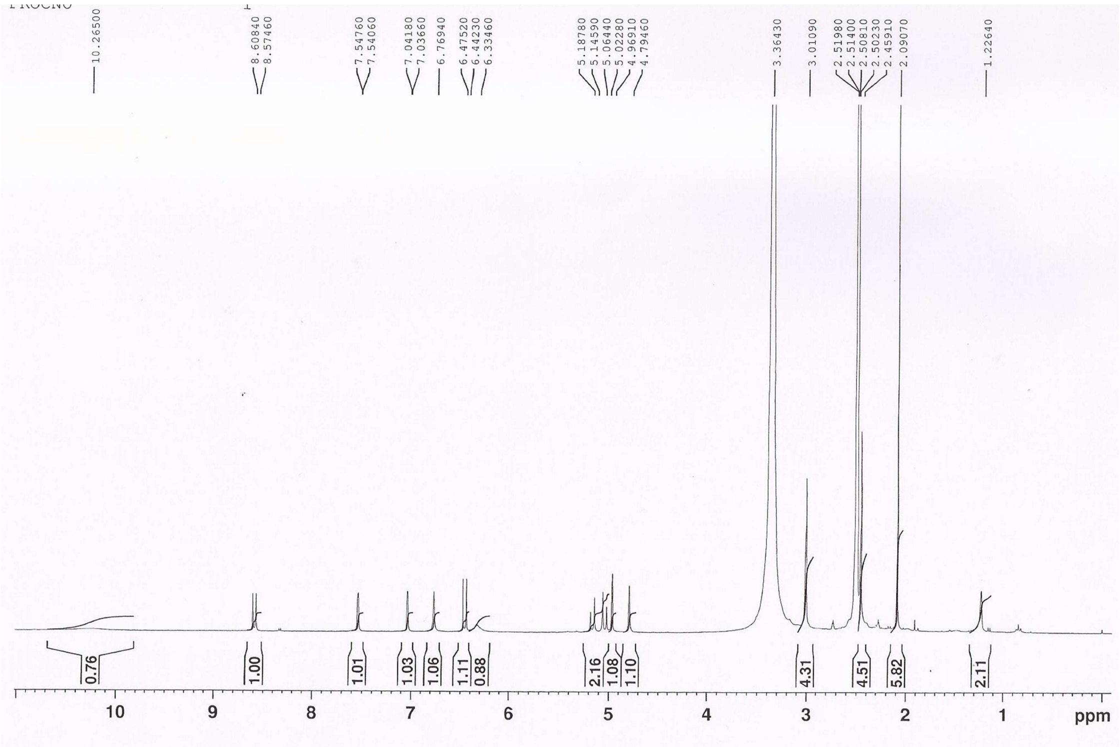

Figure S7. ^{13}C NMR spectrum of **3** (DMSO$_{d6}$,75 MHz).

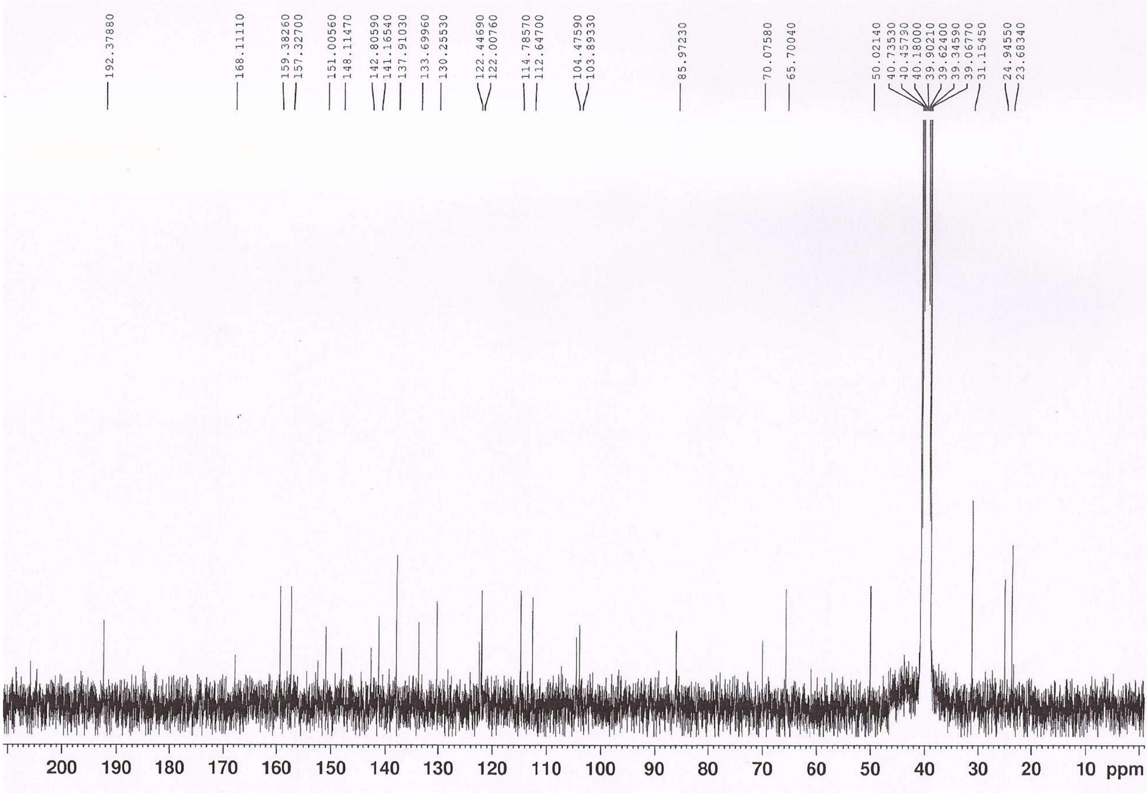

Figure S8. COSY spectrum of **3** (DMSO$_{d6}$, 300 MHz).

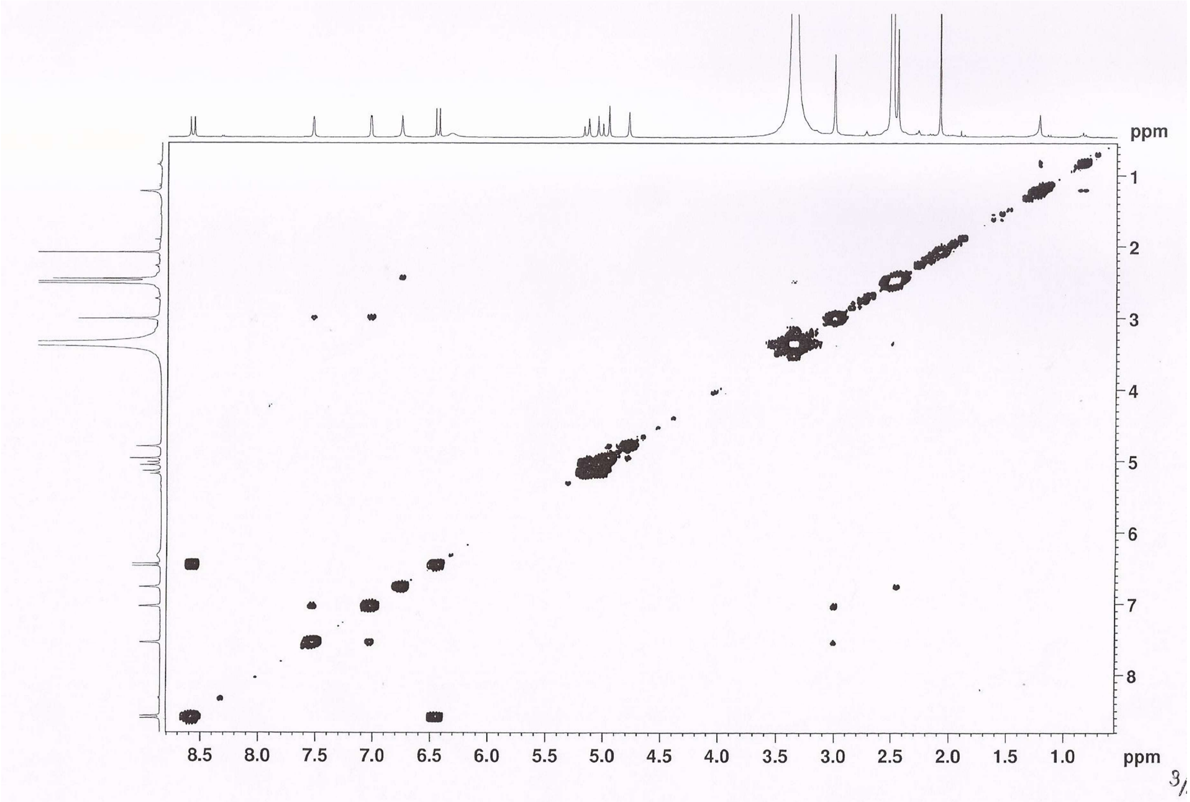

Figure S9. HSQC spectrum of **3** (DMSOd6, 300 MHz).

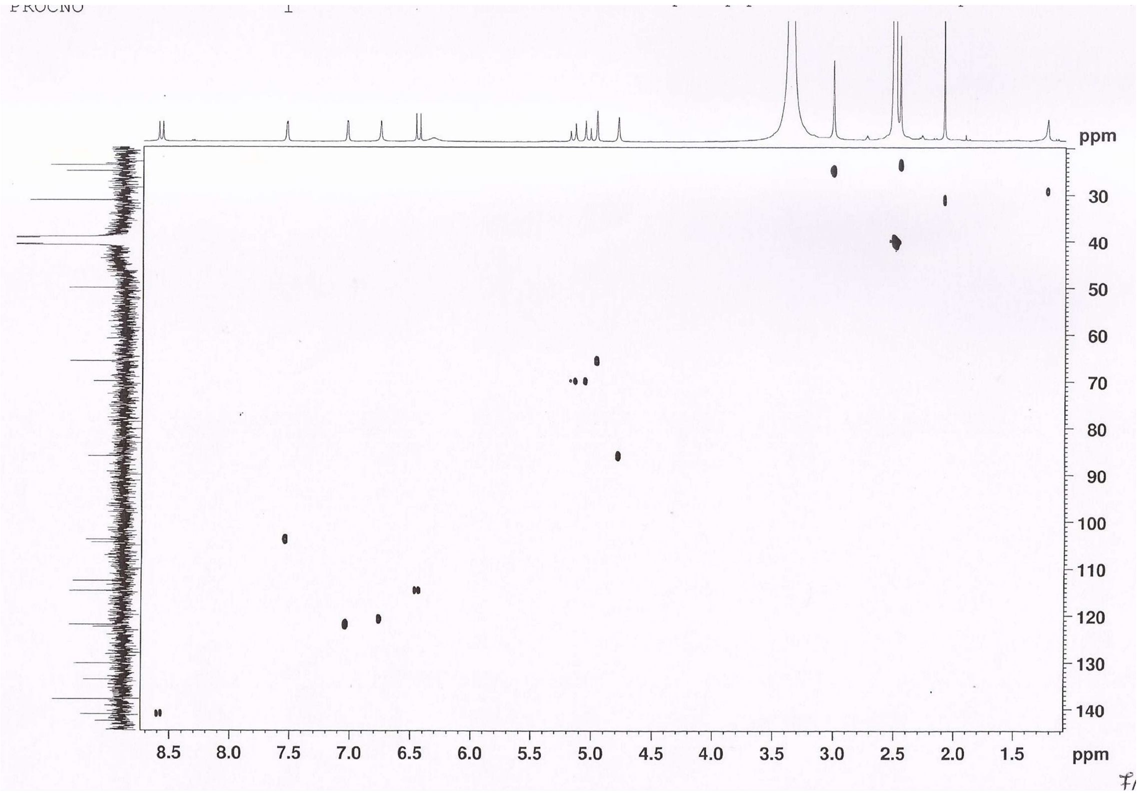

Figure S10. HMBC spectrum of **3** (DMSOd6, 300 MHz).

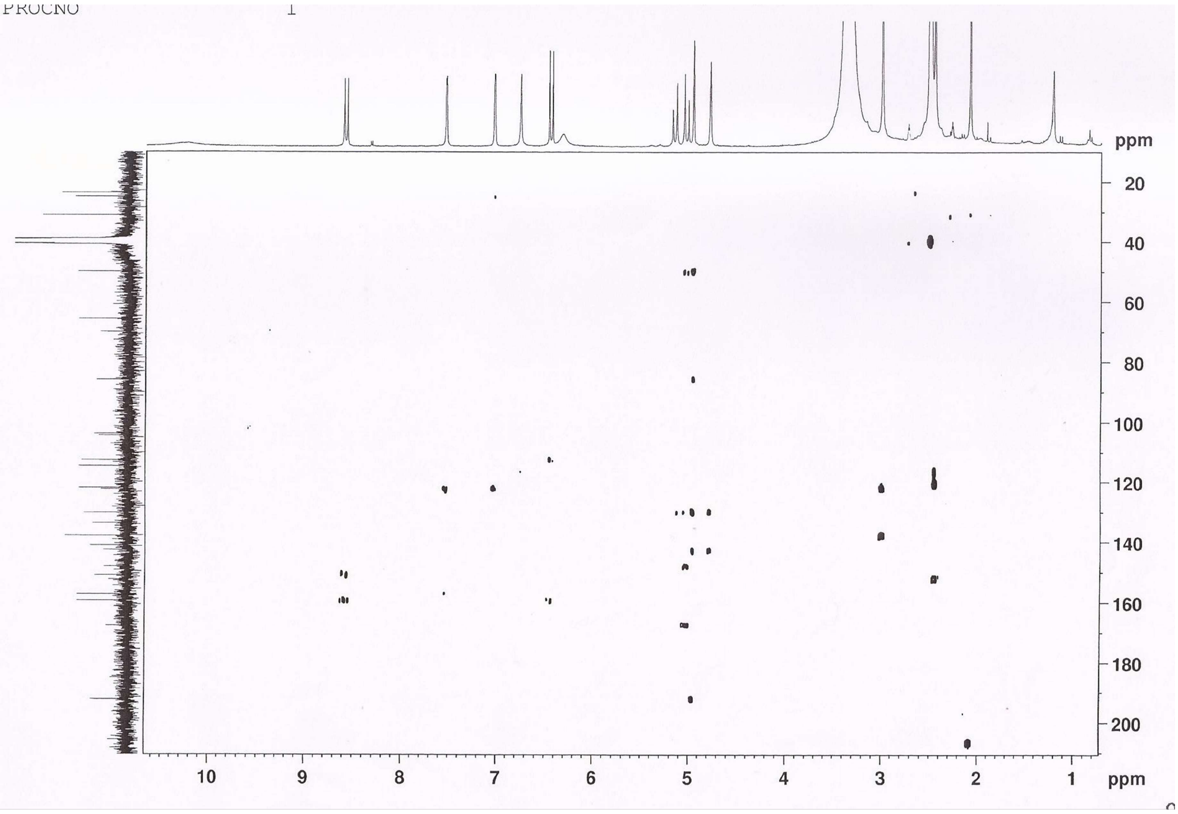

Figure S11. ROESY spectrum of **3** ($DMSO_{d6}$, 300 MHz).

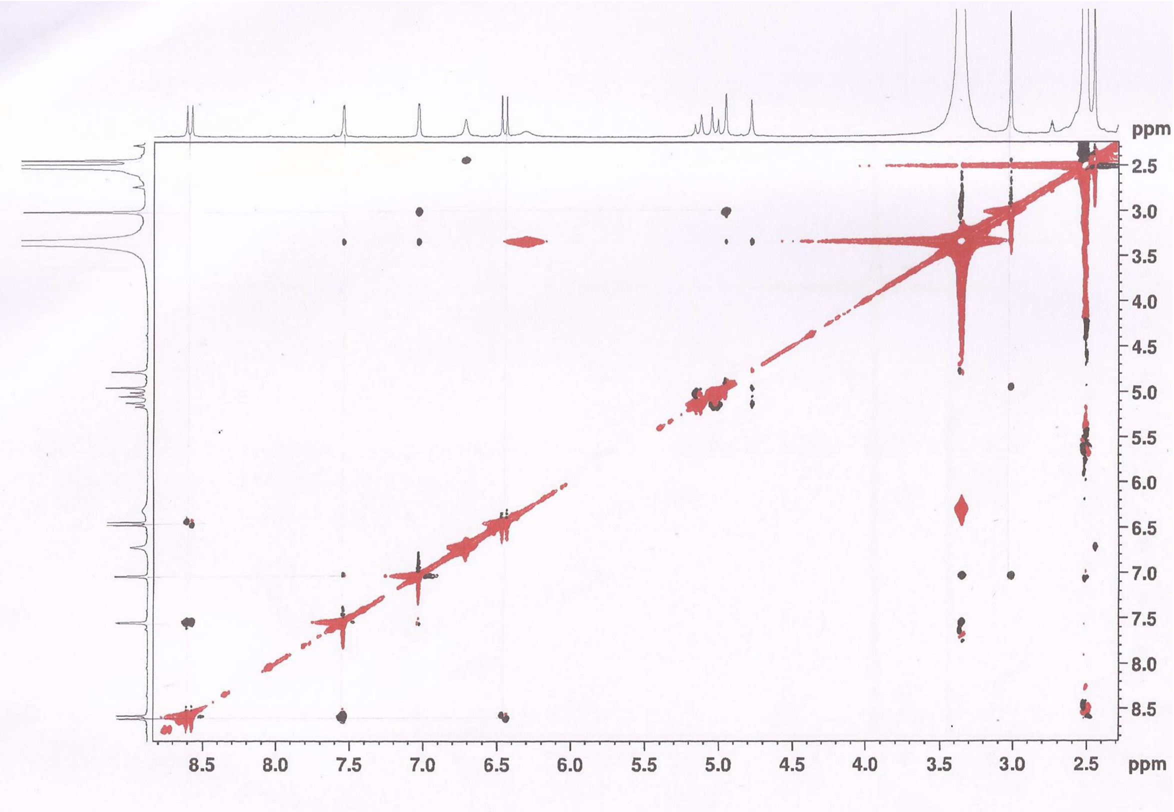

Figure S12. ^{1}H NMR spectrum of **4** ($DMSO_{d6}$, 300 MHz).

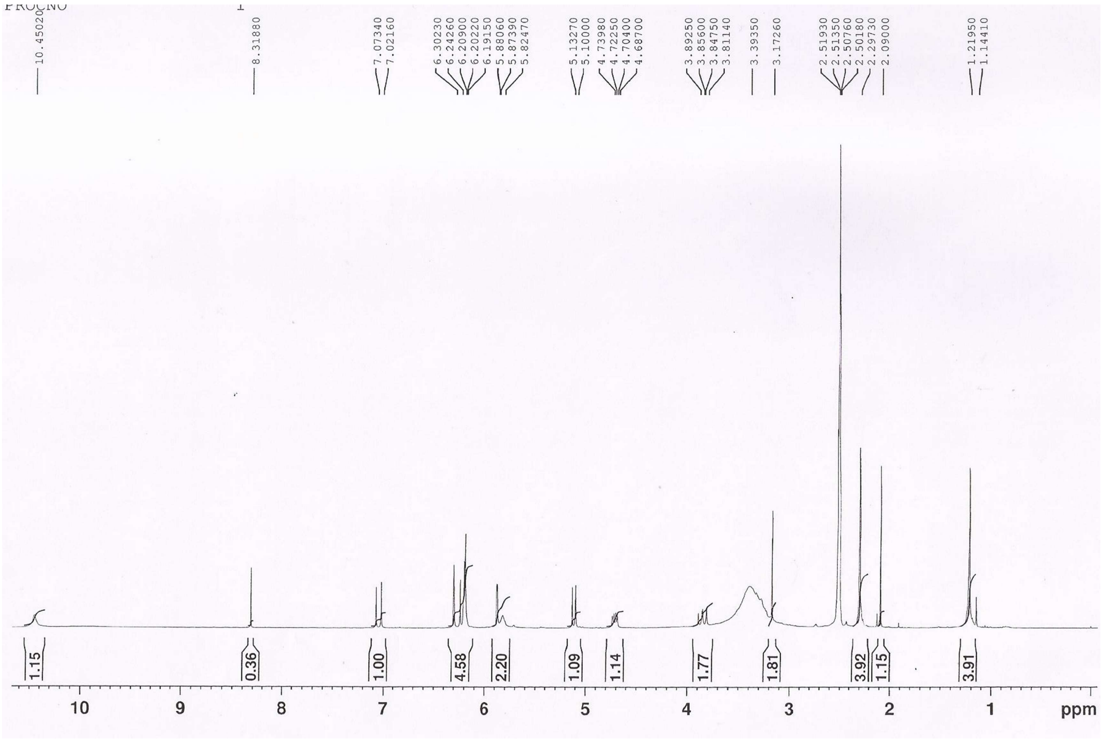

Figure S13. ^{13}C NMR spectrum of **4** (DMSO$_{d6}$, 75 MHz).

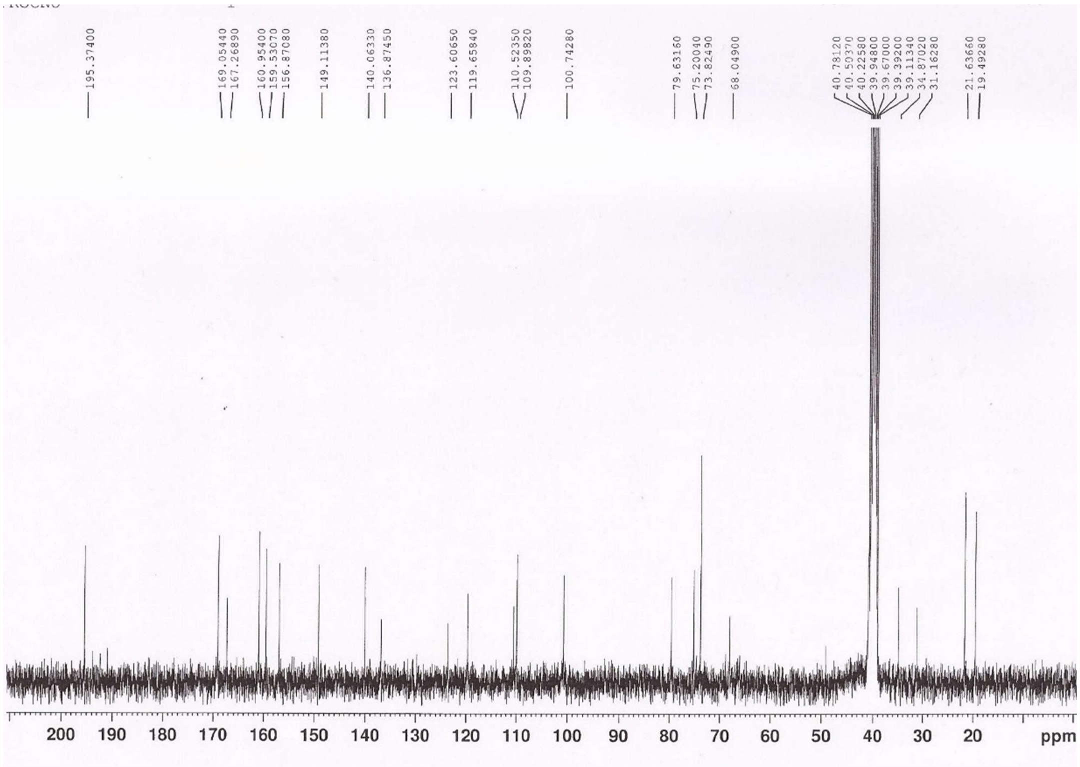

Figure S14. COSY spectrum of **4** (DMSO$_{d6}$, 300 MHz).

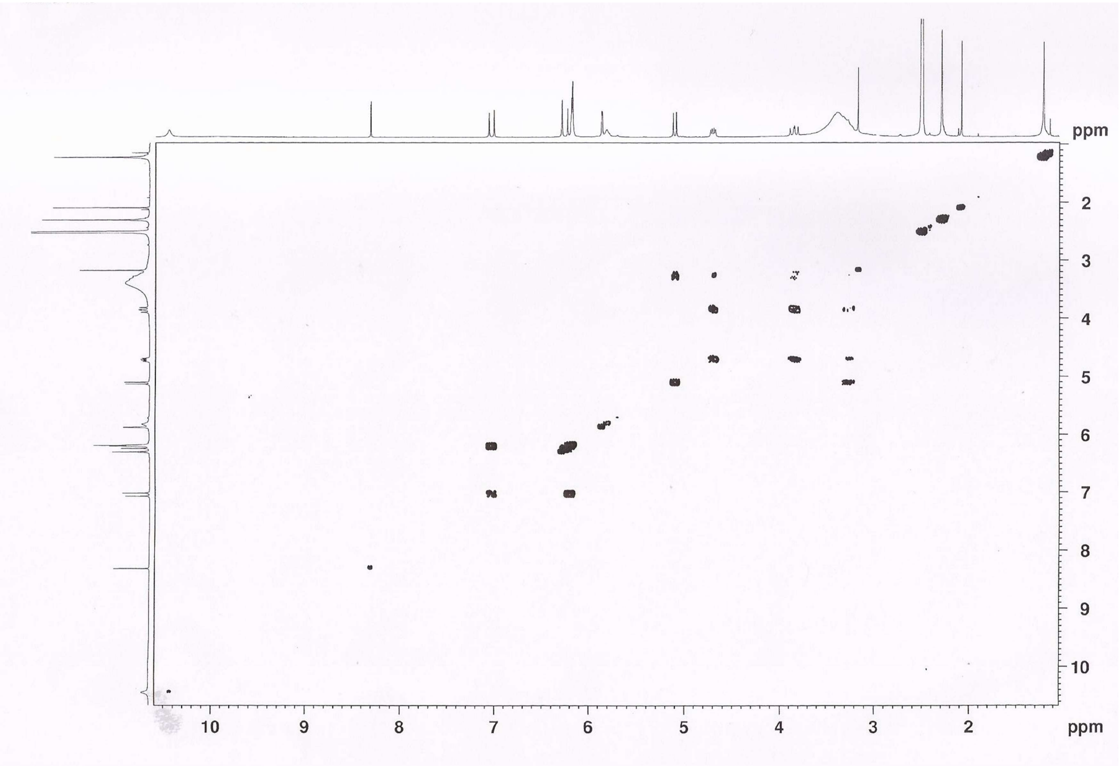

Figure S15. HSQC spectrum of **4**(DMSO$_{d6}$, 300 MHz).

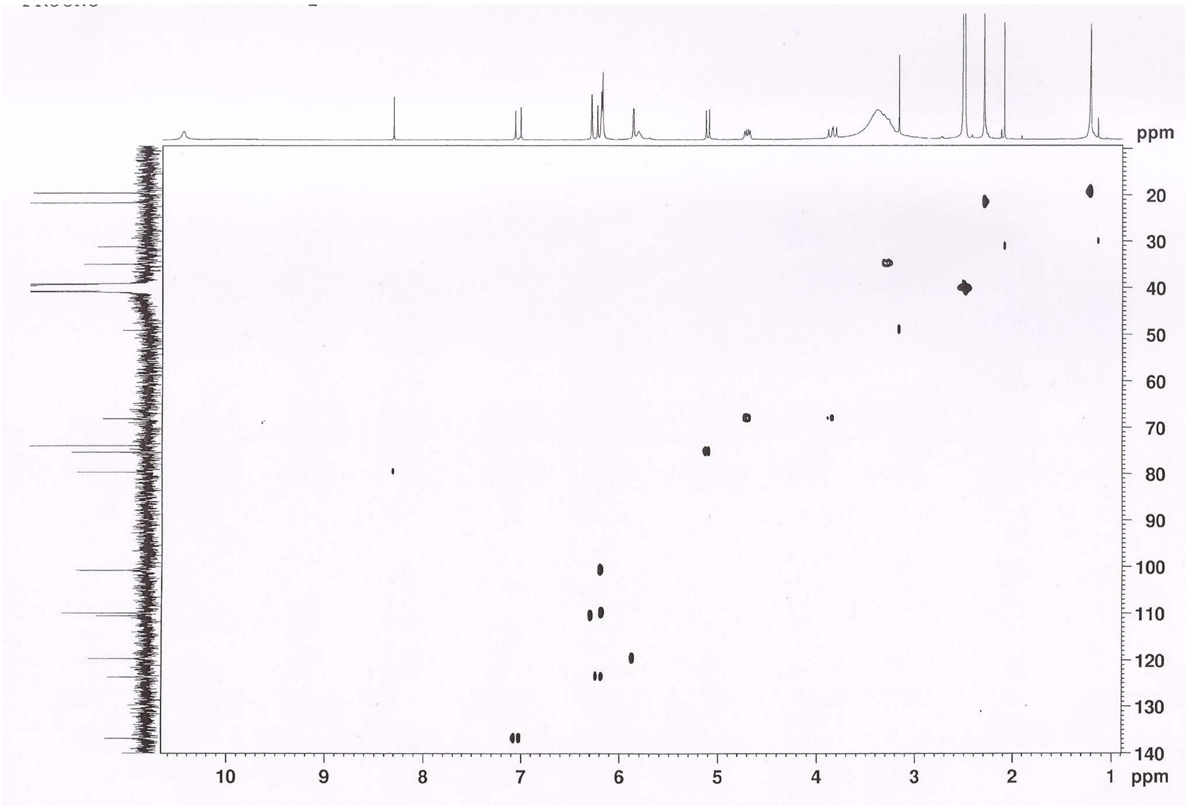

Figure S16. HMBC spectrum of **4** (DMSO$_{d6}$, 300 MHz).

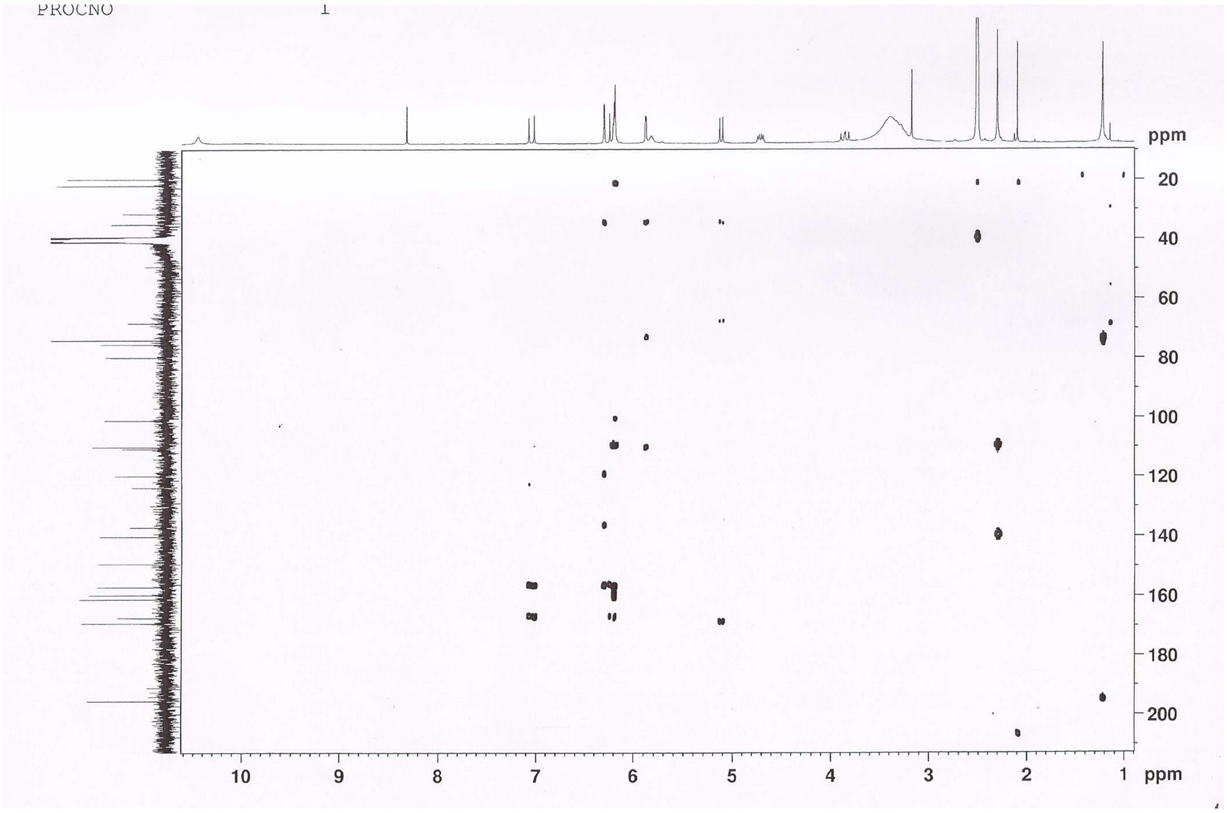

Figure S17. ROESY spectrum of **4** (DMSO$_{d6}$, 300 MHz).

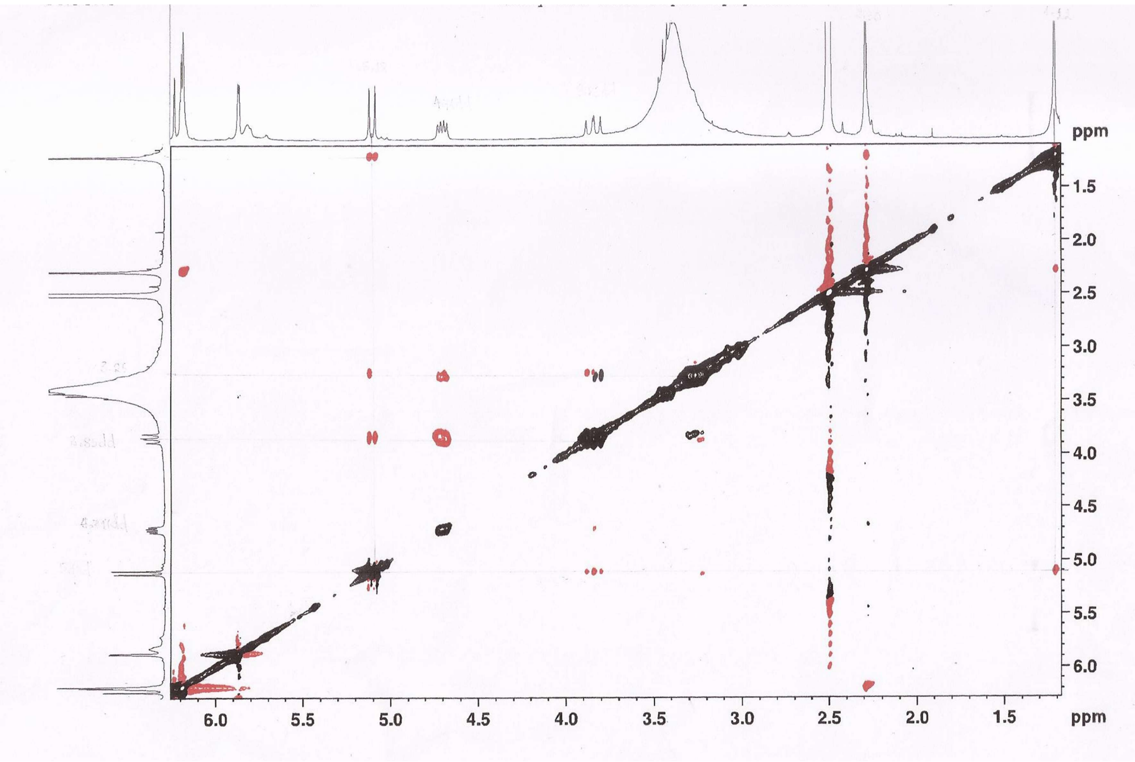

Figure S18. ^{1}H spectrum of **5** (DMSO$_{d6}$, 300 MHz).

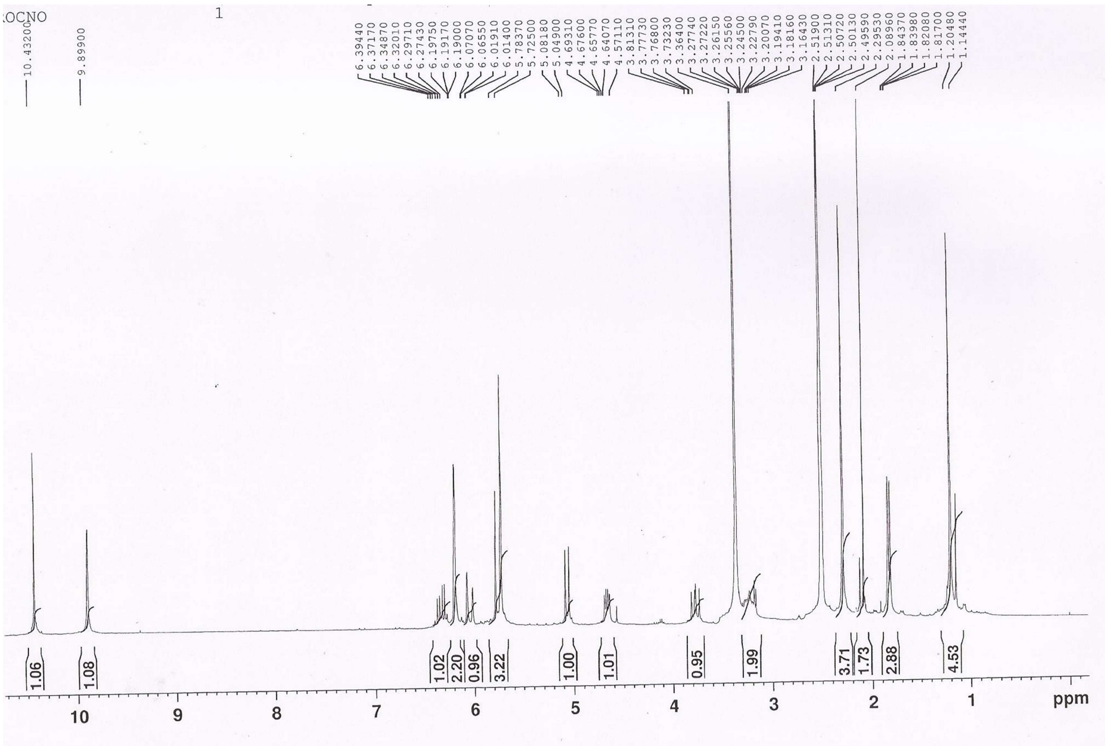

Figure S19. ^{13}C spectrum of **5** (DMSO$_{d6}$, 75 MHz).

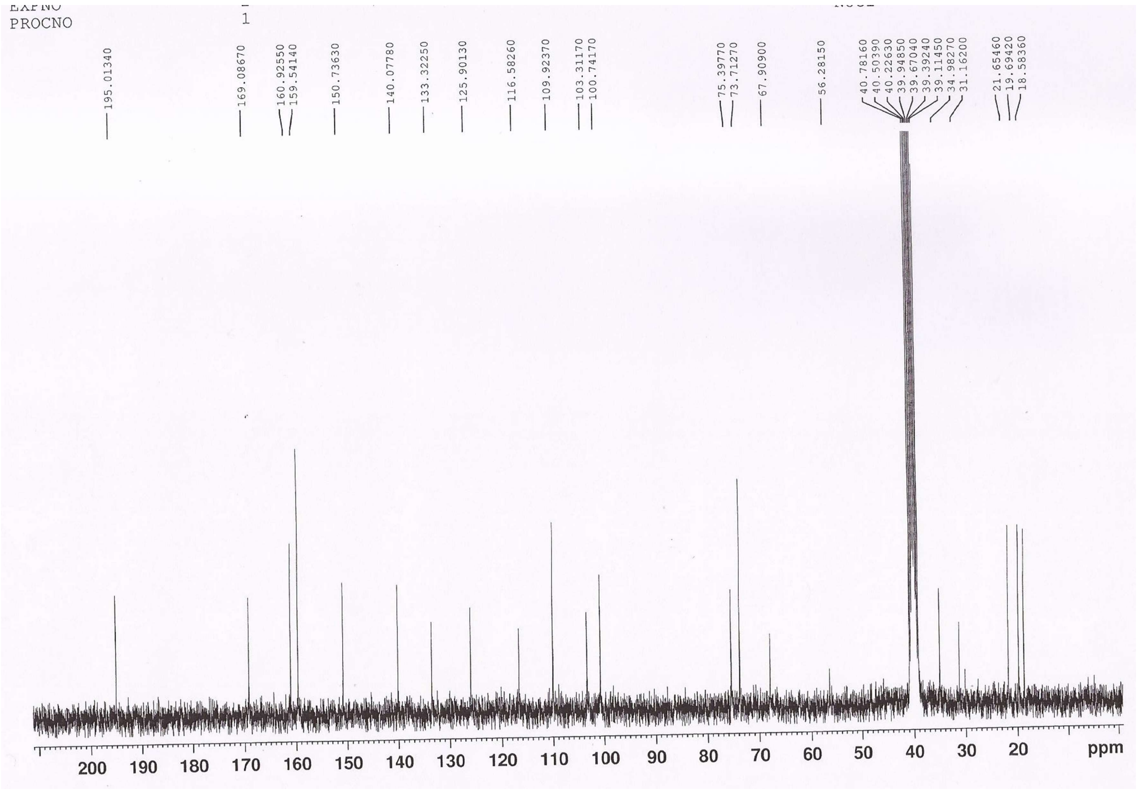

Figure S20. ROESY spectrum of **5** (DMSO$_{d6}$, 300 MHz).

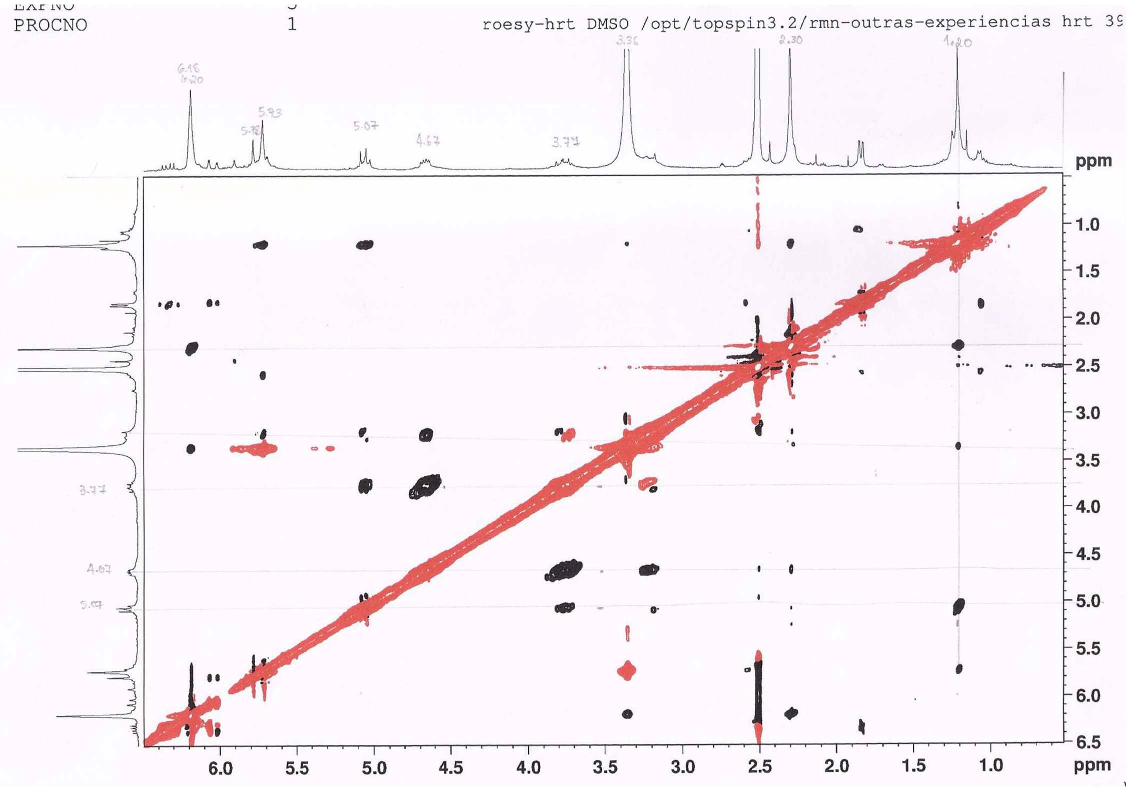

Figure S21. ^{1}H spectrum of **6** (DMSO$_{d6}$, 300 MHz).

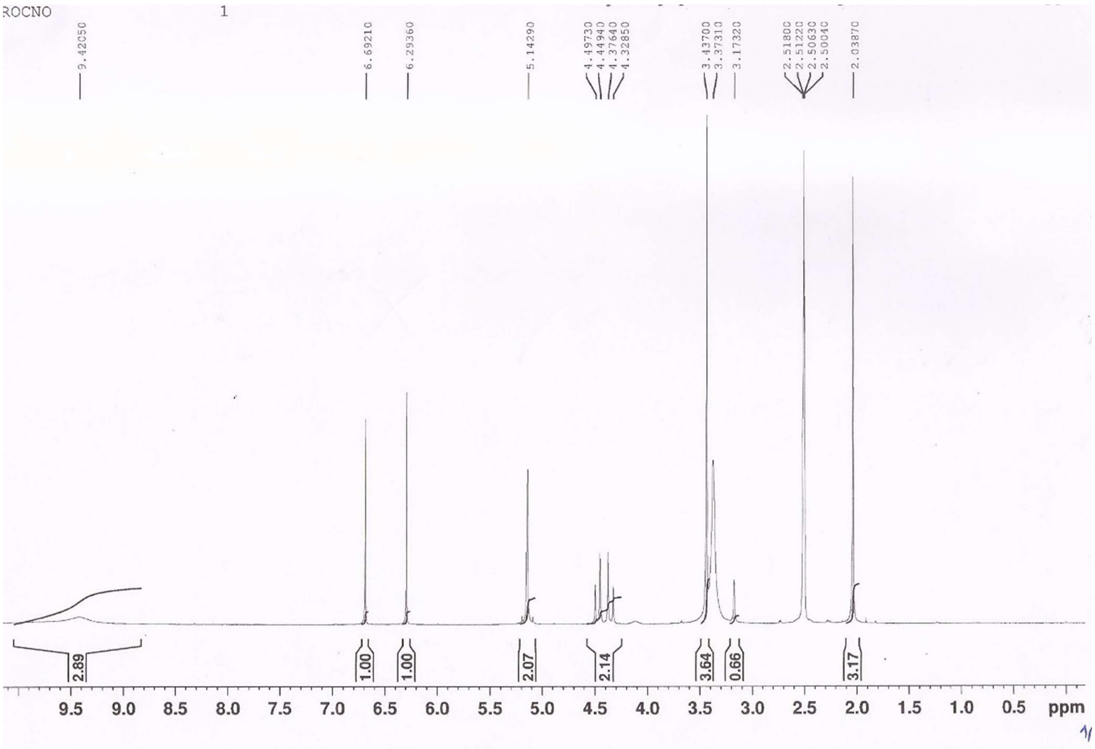

Figure S22. ^{13}C spectrum of **6** (DMSO$_{d6}$, 300 MHz).

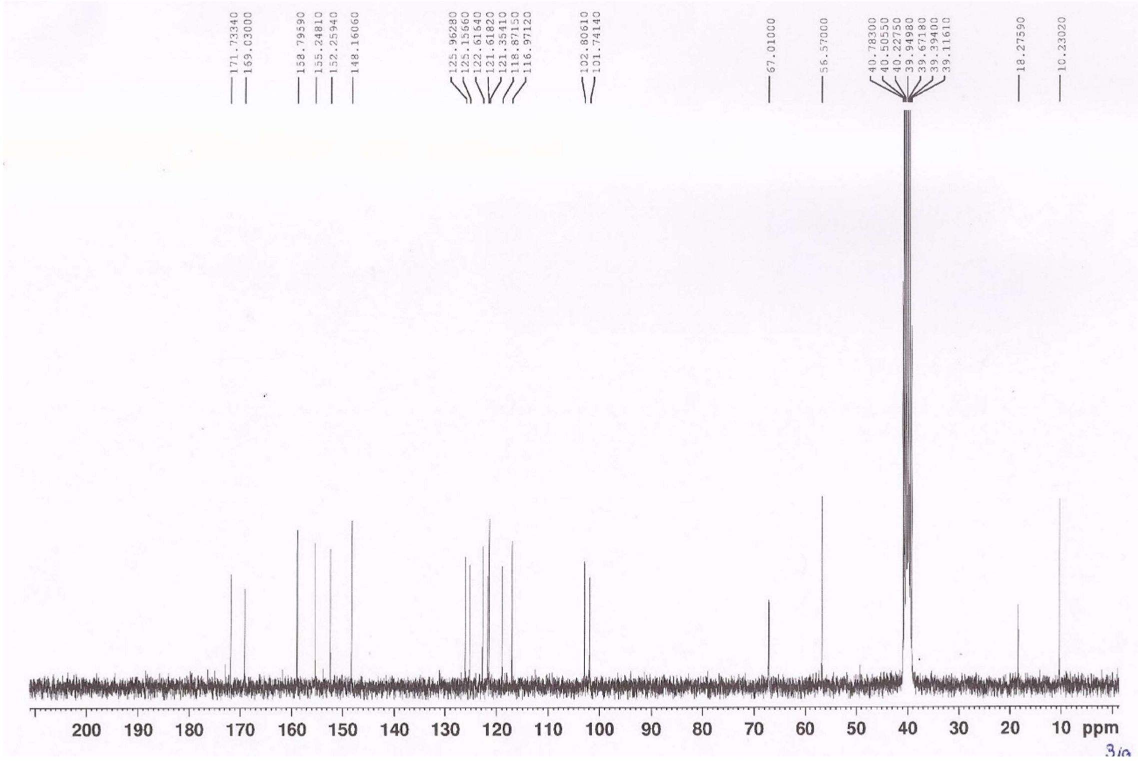

Figure S23. COSY spectrum of **6** (DMSO$_{d6}$, 300 MHz).

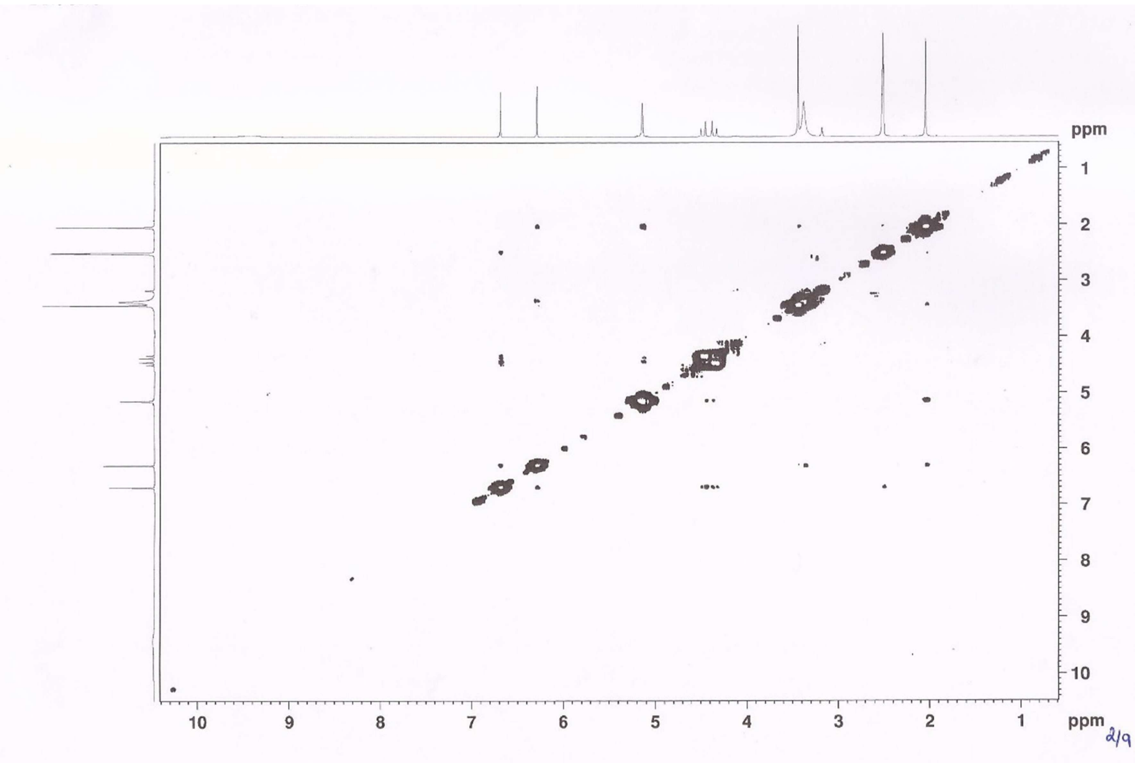

Figure S24. HSQC spectrum of **6** (DMSO$_{d6}$, 300 MHz).

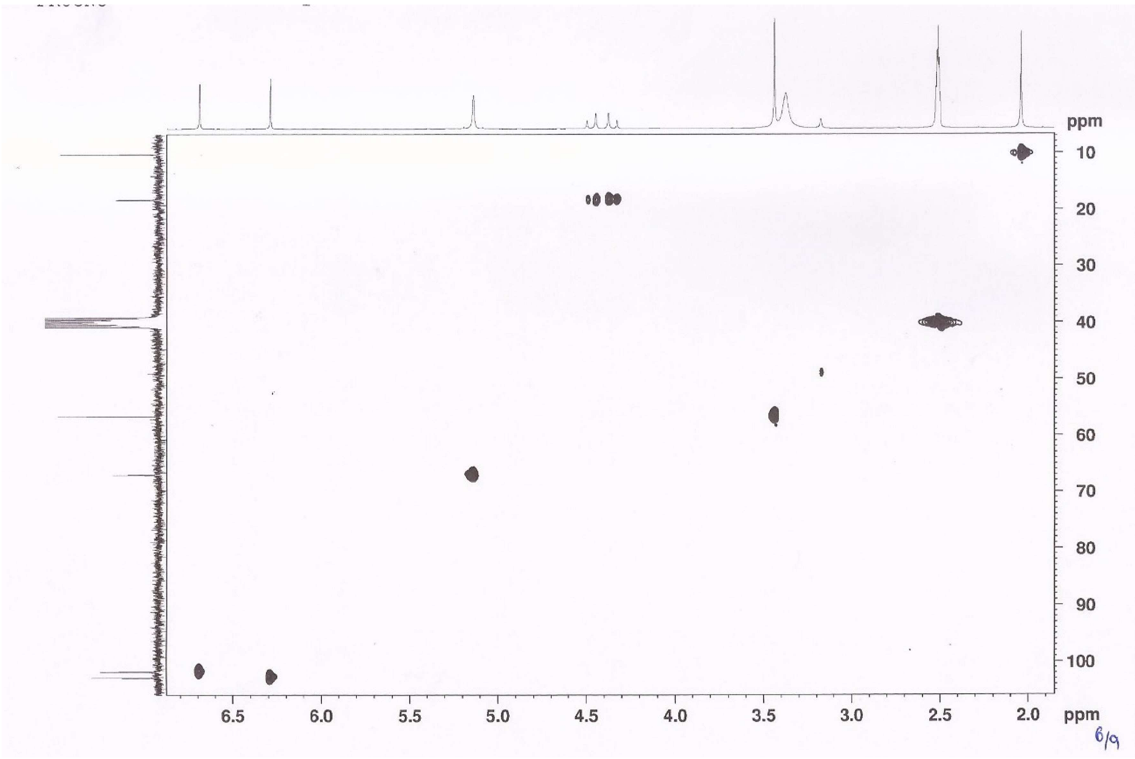

Figure S25. HMBC spectrum of **6** ($DMSO_{d6}$, 300 MHz).

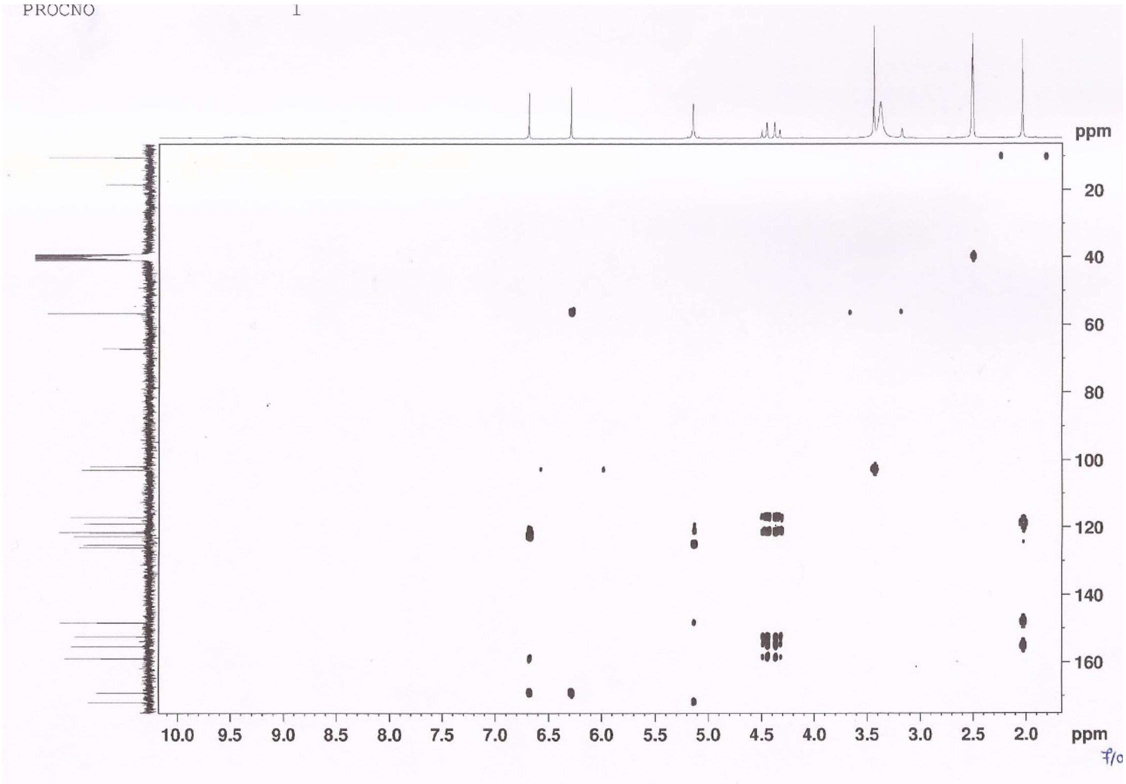

Figure S26. ^{1}H spectrum of **7** ($DMSO_{d6}$, 300 MHz).

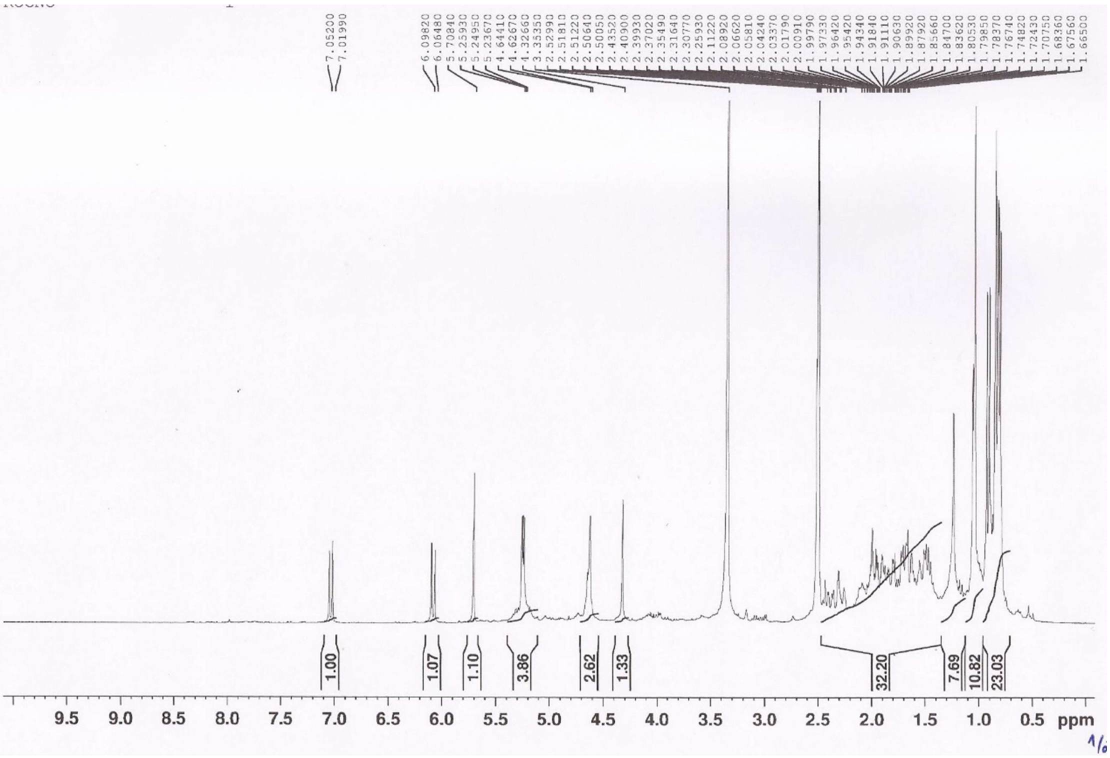

Figure S27. ^{13}C spectrum of **7** ($DMSO_{d6}$, 75 MHz).

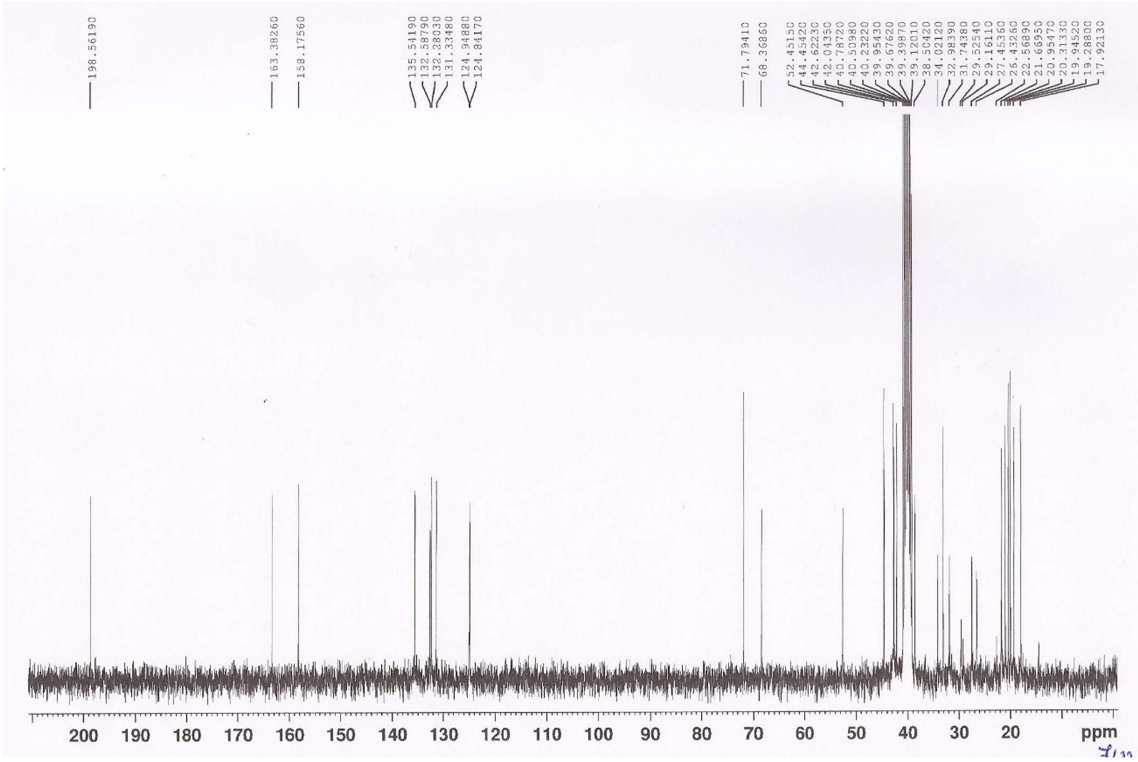

Figure S28. COSY spectrum of **7** ($DMSO_{d6}$, 300 MHz).

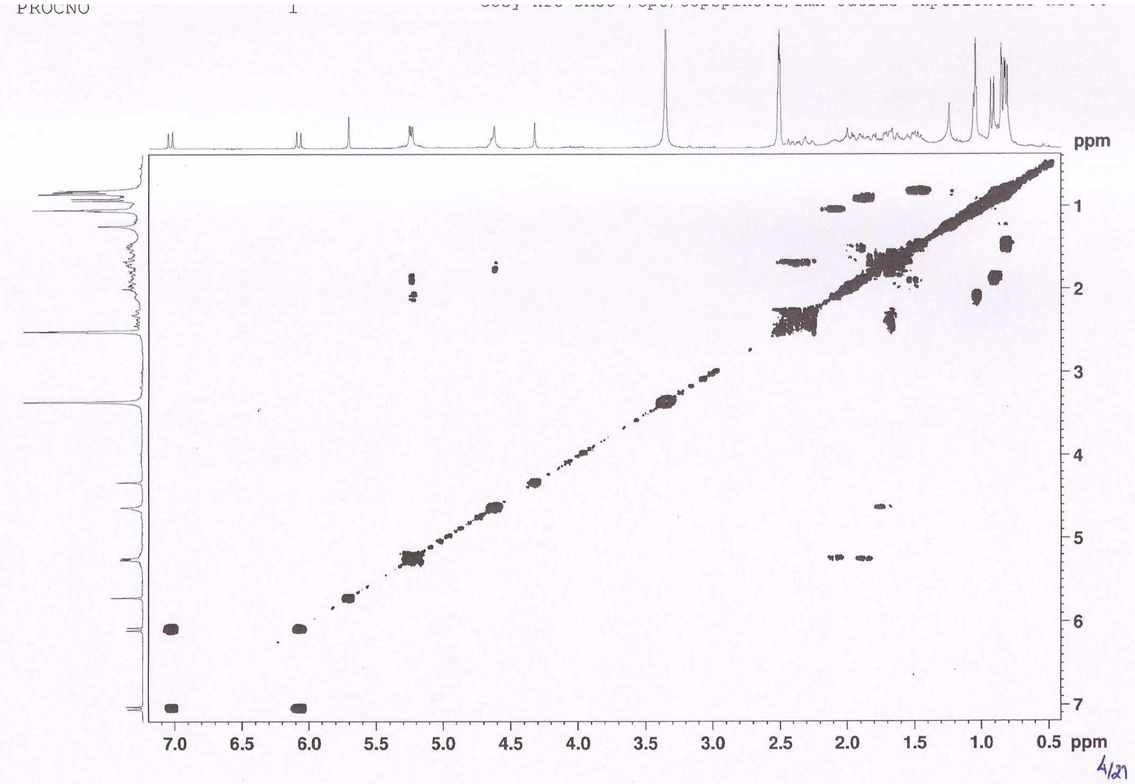

Figure S29. HSQC spectrum of **7** (DMSO$_{d6}$, 300 MHz).

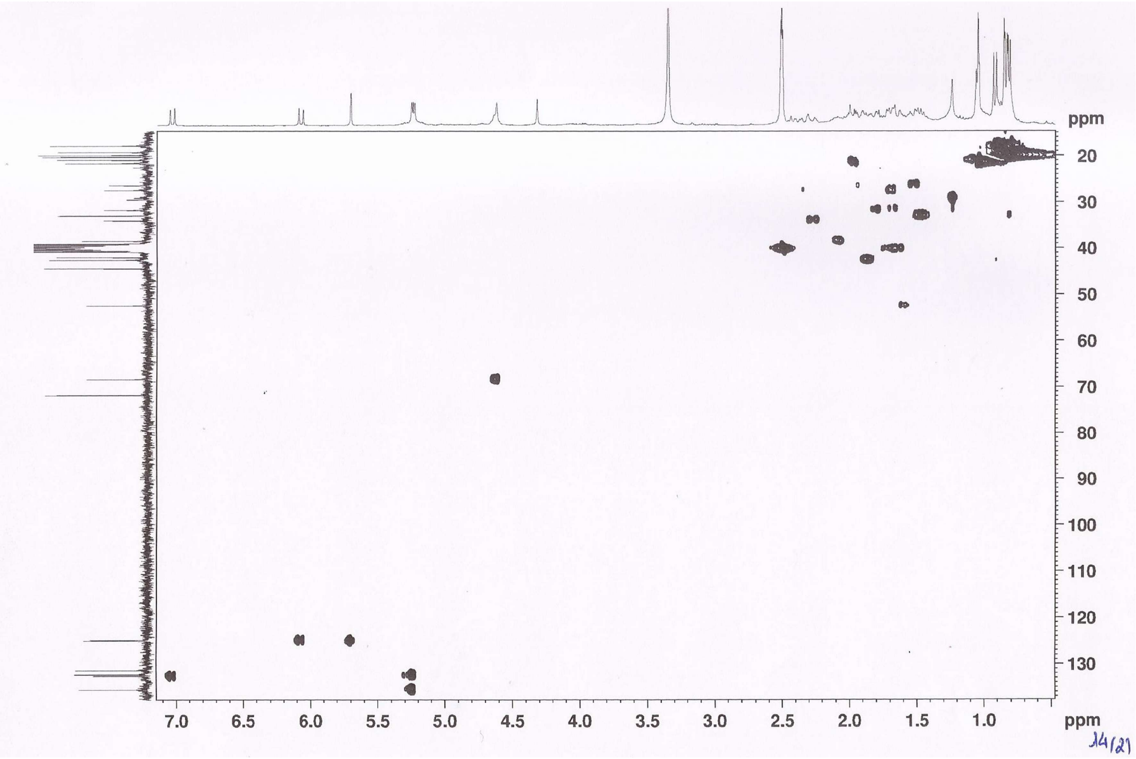

Figure S30. HMBC spectrum of **7** (DMSO$_{d6}$, 300 MHz).

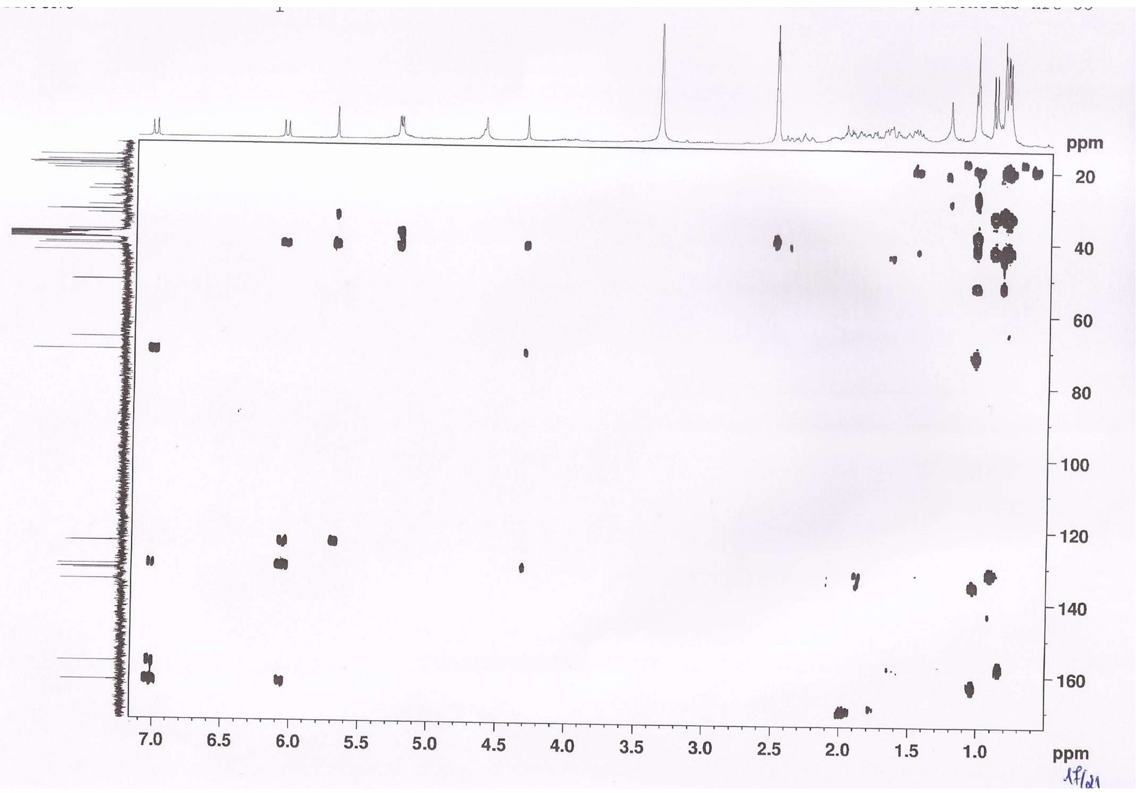

Figure S31. ^{1}H spectrum of **8** ($CDCl_3$, 300 MHz).

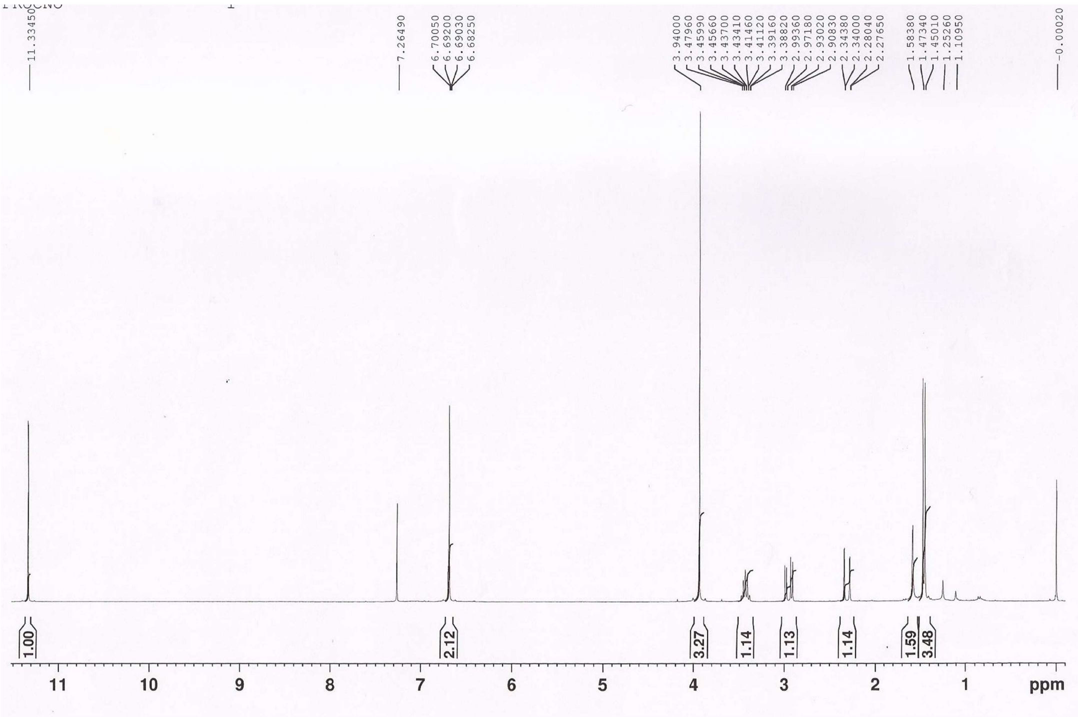

Figure S32. ^{13}C spectrum of **8** ($CDCl_3$, 75MHz).

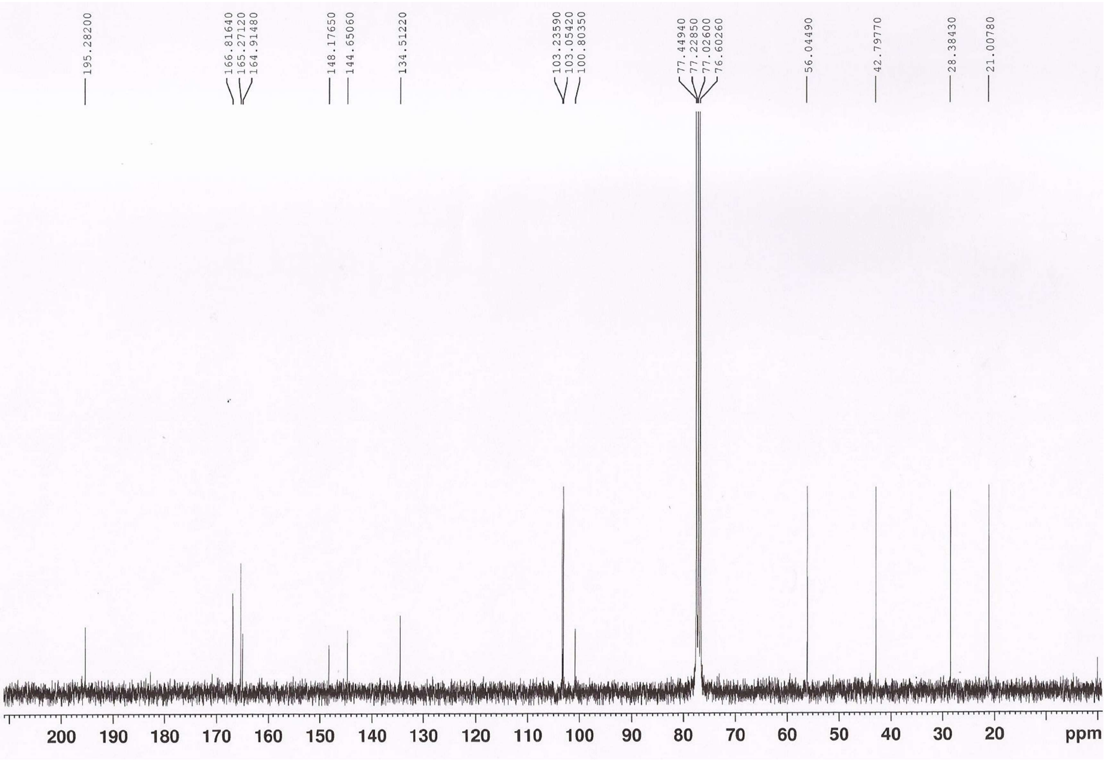

Figure S33. COSY spectrum of **8** ($CDCl_3$, 300 MHz).

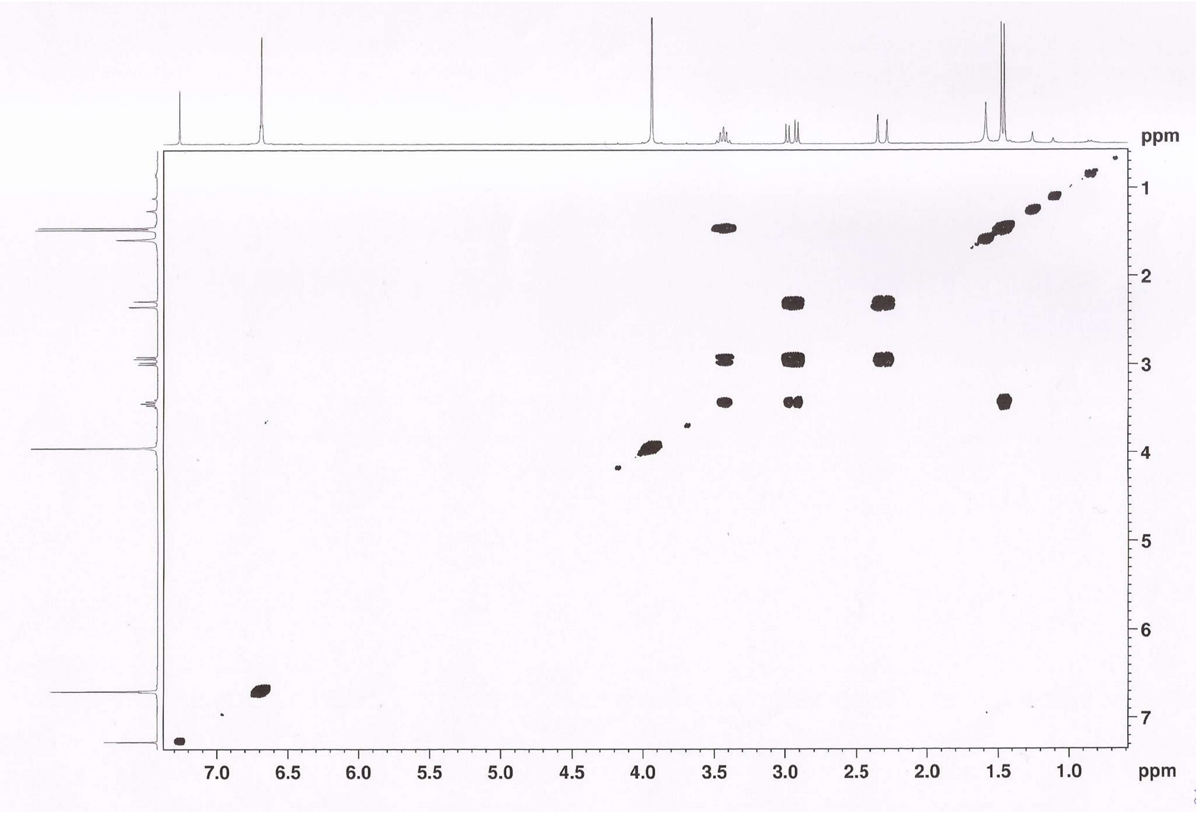

Figure S34. HSQC spectrum of **8** ($CDCl_3$, 300 MHz).

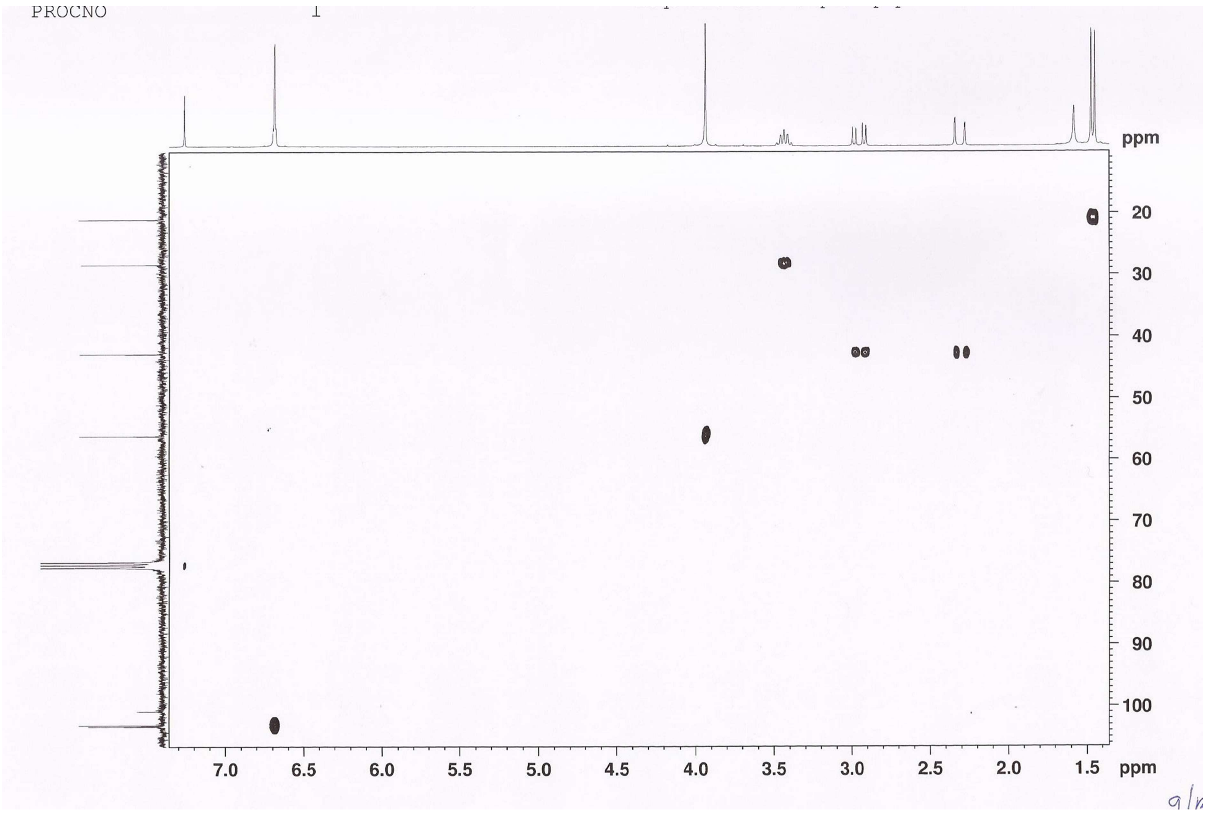

Figure S35. HMBC spectrum of **8** ($CDCl_3$, 300 MHz).

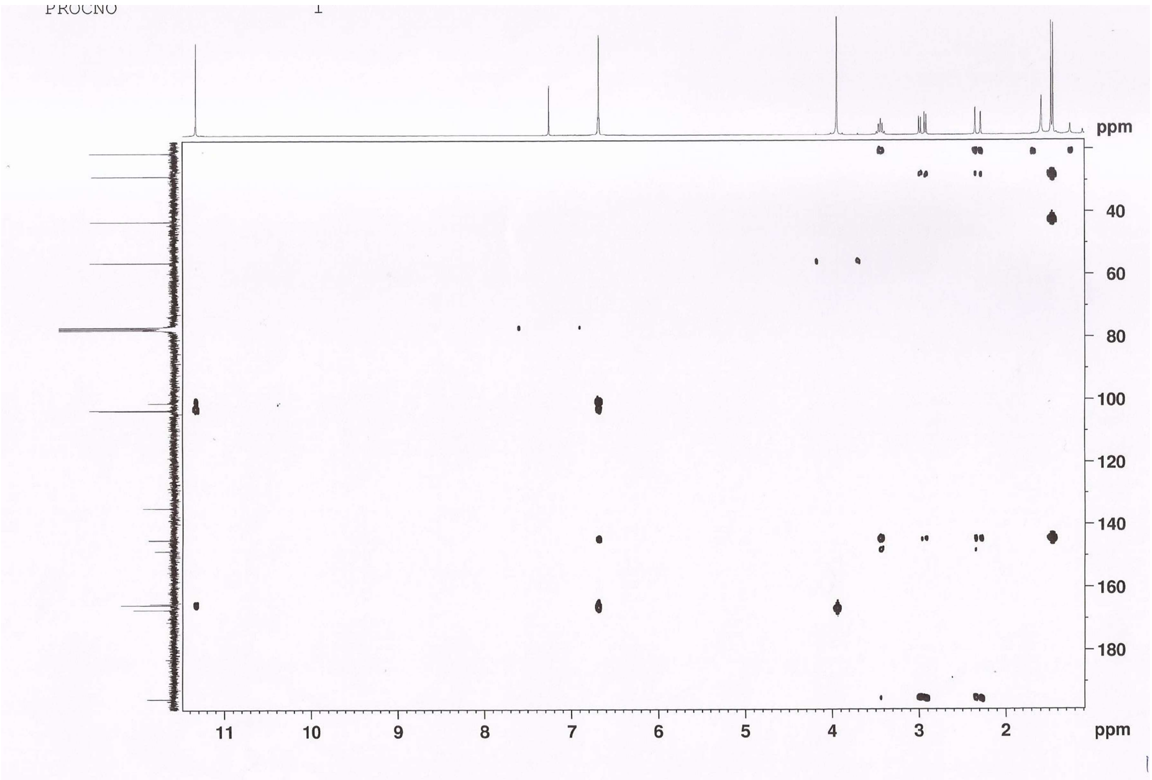

Figure S36. ^{1}H spectrum of **9** (DMSO$_{d6}$, 500 MHz).

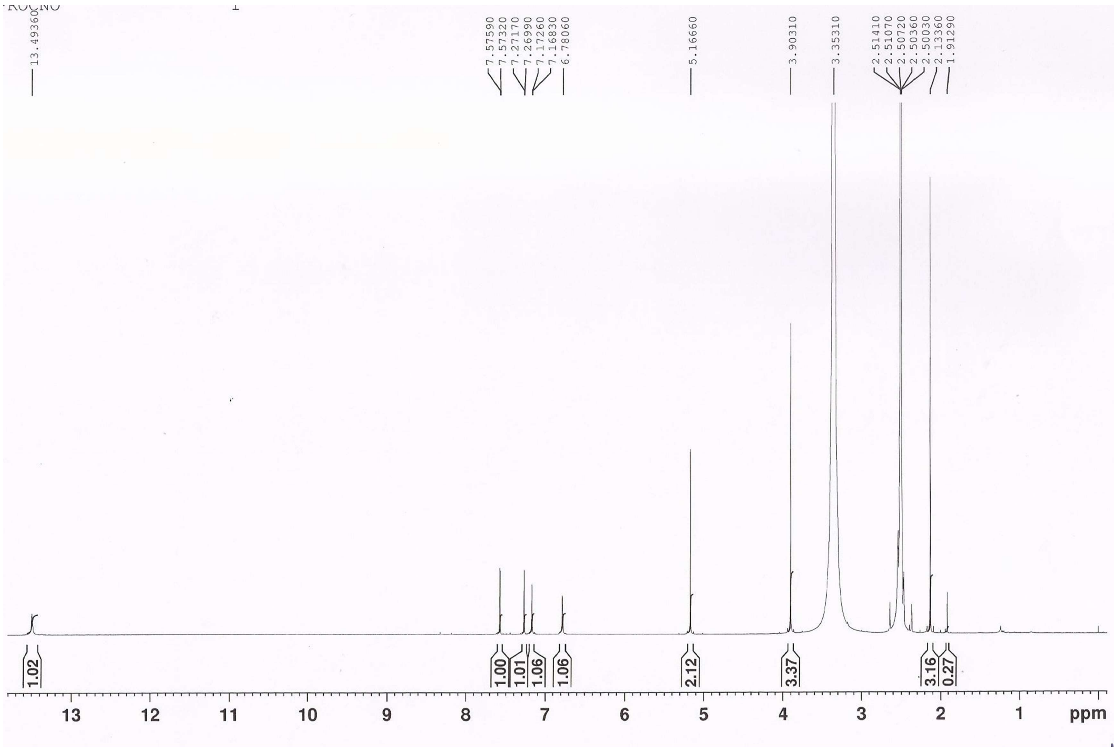

Figure S37. ^{13}C spectrum of **9** (DMSO$_{d6}$, 125MHz).

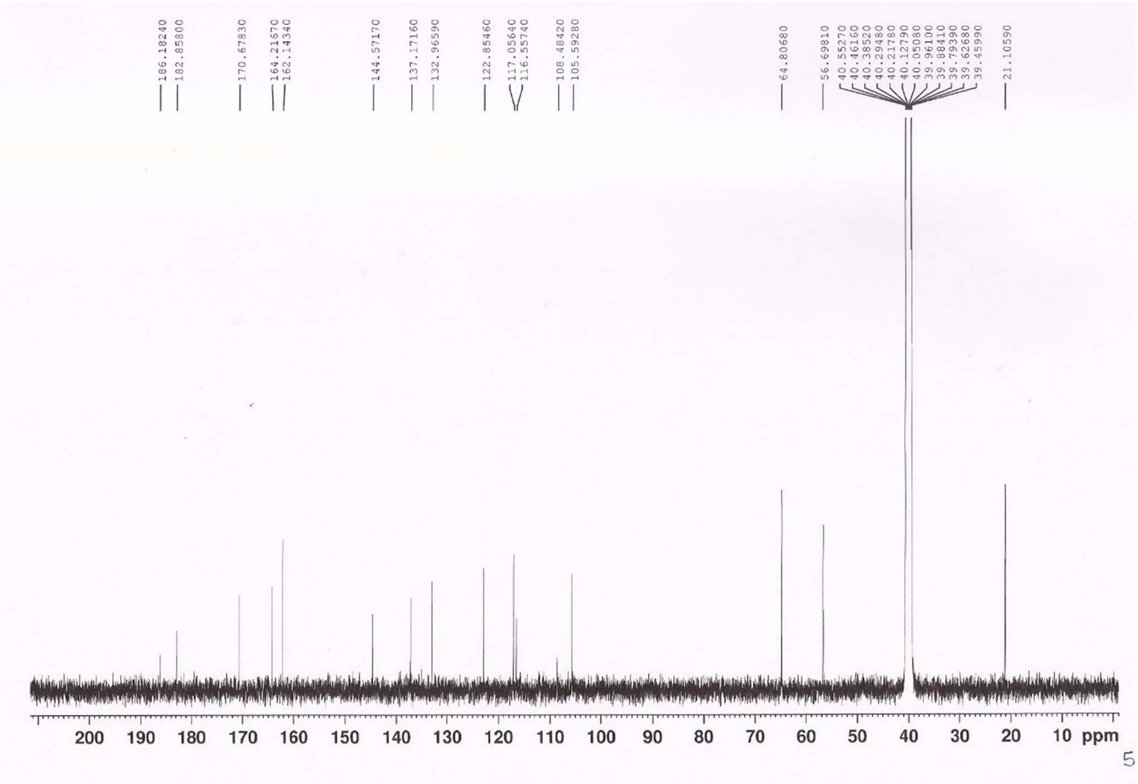

Figure S38. ^{1}H spectrum of **10** (DMSO$_{d6}$, 300 MHz).

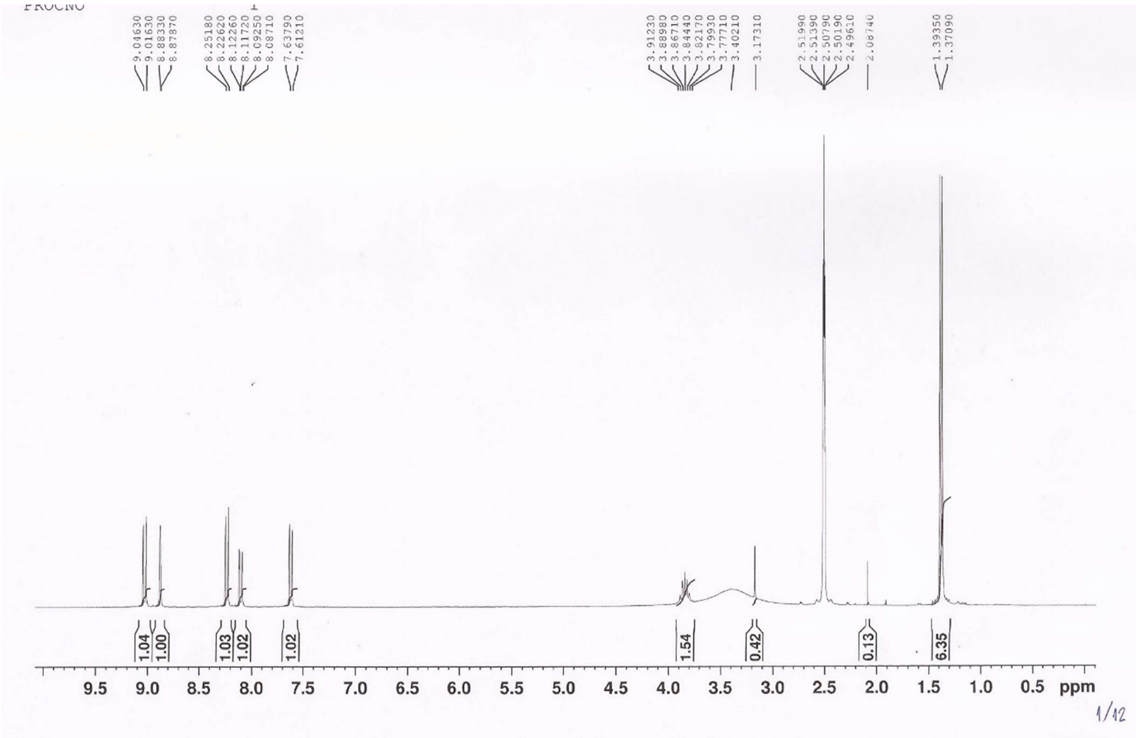

Figure S39. ^{13}C spectrum of **10** (DMSO$_{d6}$, 75 MHz).

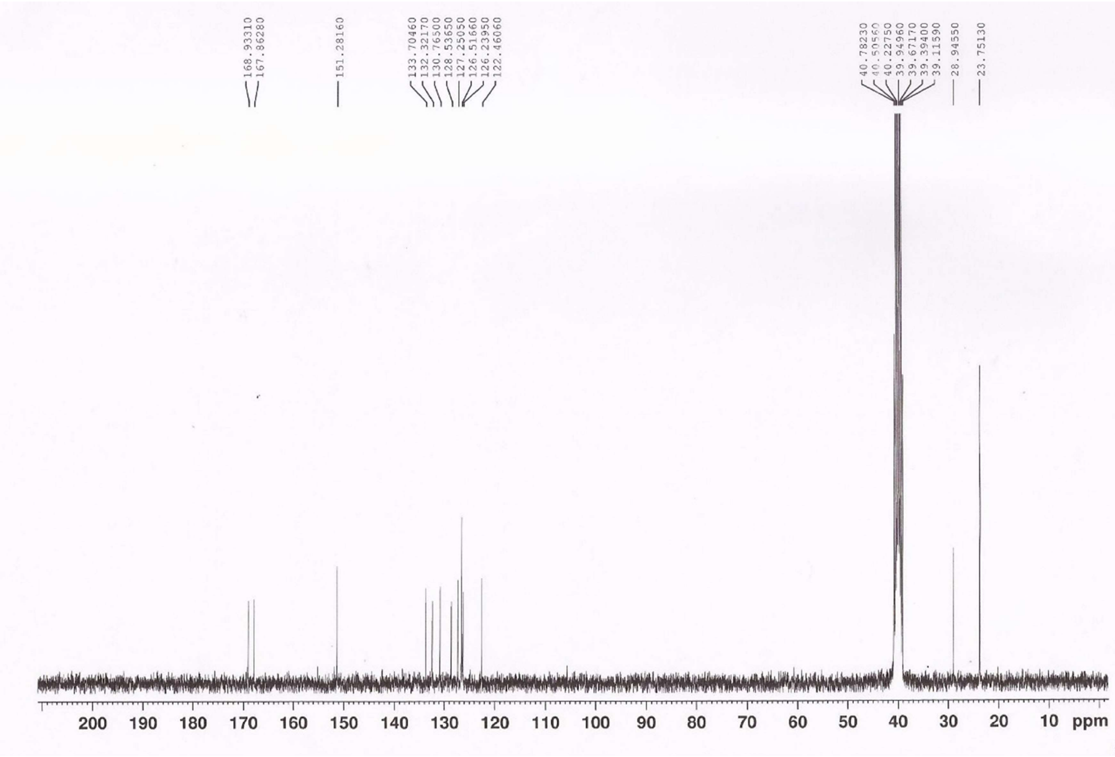

Figure S40. ^{1}H spectrum of **11** ($DMSO_{d6}$, 300 MHz).

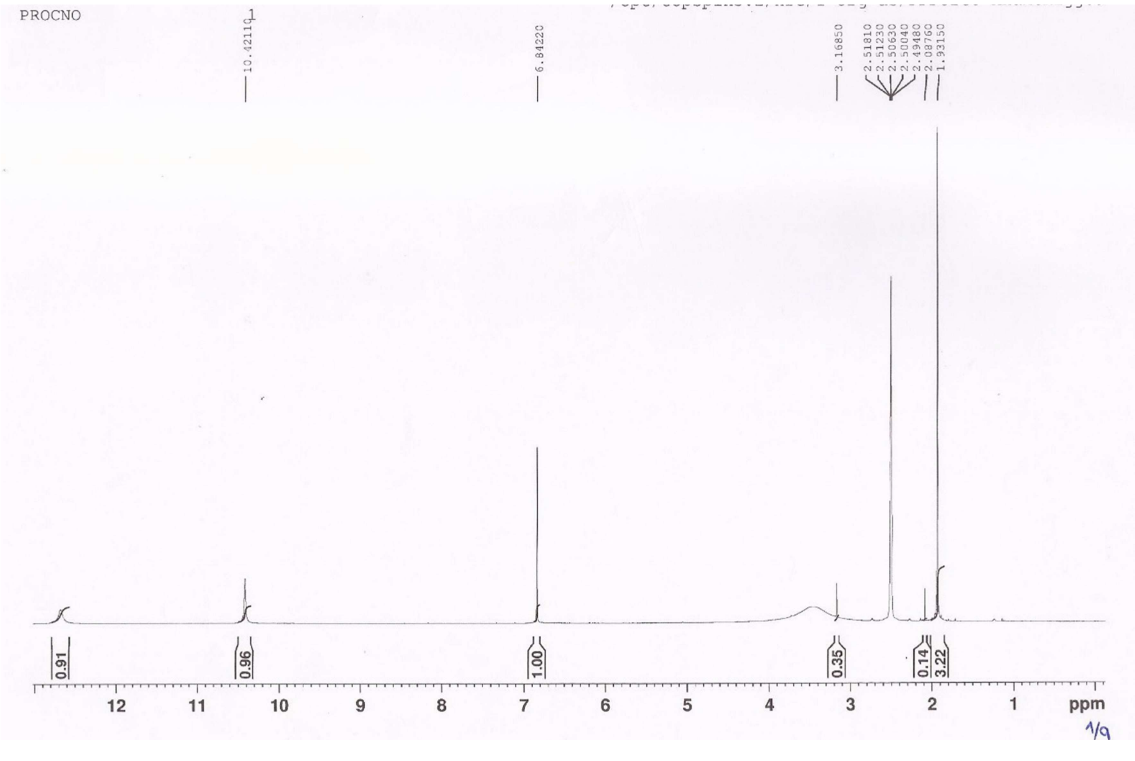

Figure S41. ^{13}C spectrum of **11** ($DMSO_{d6}$, 75 MHz).

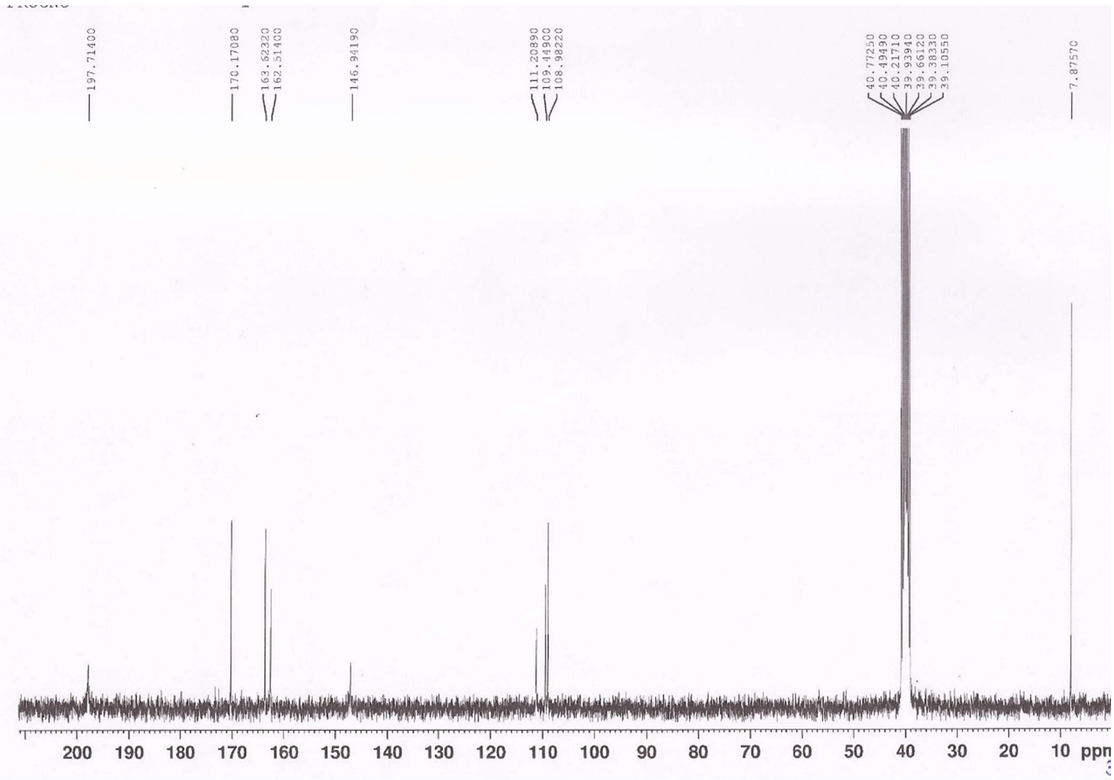

Figure S42. (+)-HRESIMS of **3**

Elemental Composition Report [MH]$^+$

Single Mass Analysis
Tolerance = 5.0 PPM / DBE: min = -1.5, max = 100.0
Element prediction: Off
Number of isotope peaks used for i-FIT = 3
Monoisotopic Mass, Even Electron Ions
30 formula(e) evaluated with 1 results within limits (up to 100 closest results for each mass)
Elements Used:
C: 27-27 H: 0-150 O: 0-30

Minimum:				-1.5				
Maximum:		5.0	5.0	100.0				
Mass	Calc. Mass	mDa	PPM	DBE	i-FIT	Norm	Conf(%)	Formula
471.1075	471.1080	-0.5	-1.1	18.5	778.2	n/a	n/a	C27 H19 O8

Elemental Composition Report [MNa]$^+$

Single Mass Analysis
Tolerance = 5.0 PPM / DBE: min = -1.5, max = 100.0
Element prediction: Off
Number of isotope peaks used for i-FIT = 3
Monoisotopic Mass, Even Electron Ions
61 formula(e) evaluated with 1 results within limits (up to 100 closest results for each mass)
Elements Used:
C: 27-27 H: 0-150 O: 0-30 Na: 0-1

Minimum:				-1.5				
Maximum:		5.0	5.0	100.0				
Mass	Calc. Mass	mDa	PPM	DBE	i-FIT	Norm	Conf(%)	Formula
493.0895	493.0899	-0.4	-0.8	18.5	752.8	n/a	n/a	C27 H18 O8 Na

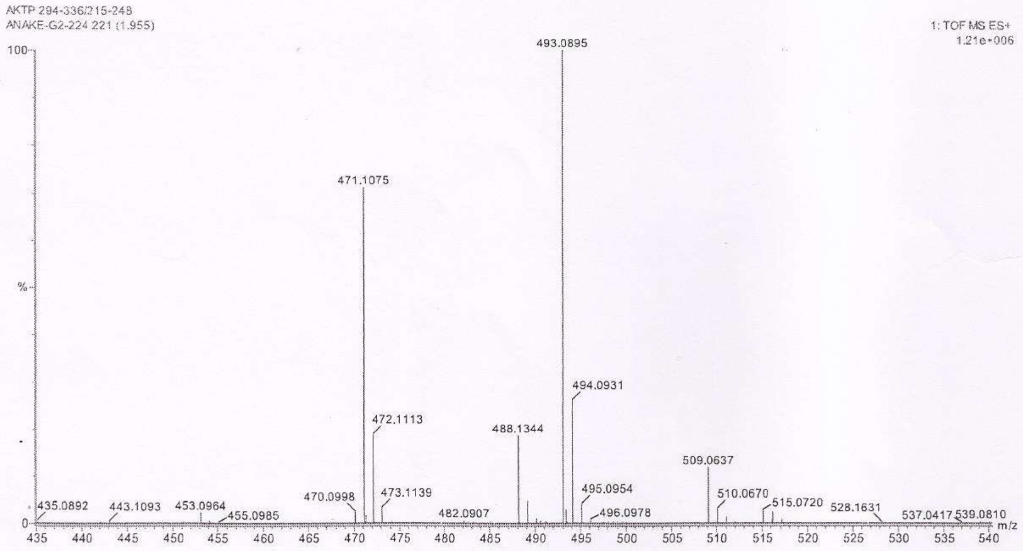

Figure S43. (+)-HRESIMS of **4**

Elemental Composition Report [MH]+

Single Mass Analysis
Tolerance = 5.0 PPM / DBE: min = -1.5, max = 100.0
Element prediction: Off
Number of isotope peaks used for i-FIT = 3

Monoisotopic Mass, Even Electron Ions
27 formula(e) evaluated with 1 results within limits (up to 100 closest results for each mass)
Elements Used:
C: 21-21 H: 0-150 O: 0-30

Minimum:				-1.5				
Maximum:		5.0	5.0	100.0				
Mass	Calc. Mass	mDa	PPM	DBE	i-FIT	Norm	Conf(%)	Formula
417.1186	417.1186	0.0	0.0	11.5	514.2	n/a	n/a	C21 H21 O9

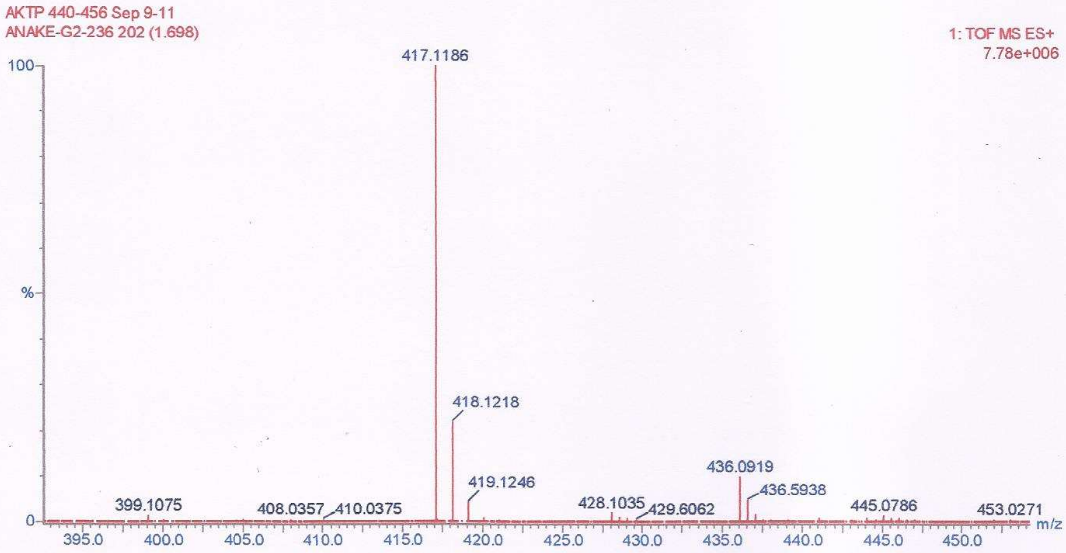

Figure S44. (+)-HRESIMS of **6**

Elemental Composition Report $[MH]^+$

Single Mass Analysis
Tolerance = 5.0 PPM / DBE: min = -1.5, max = 100.0
Element prediction: Off
Number of isotope peaks used for i-FIT = 3

Monoisotopic Mass, Even Electron Ions
25 formula(e) evaluated with 1 results within limits (up to 100 closest results for each mass)
Elements Used:
C: 19-19 H: 0-150 O: 0-30

Minimum:				-1.5				
Maximum:		5.0	5.0	100.0				
Mass	Calc. Mass	mDa	PPM	DBE	i-FIT	Norm	Conf(%)	Formula
389.0870	389.0873	-0.3	-0.8	11.5	410.3	n/a	n/a	C19 H17 O9

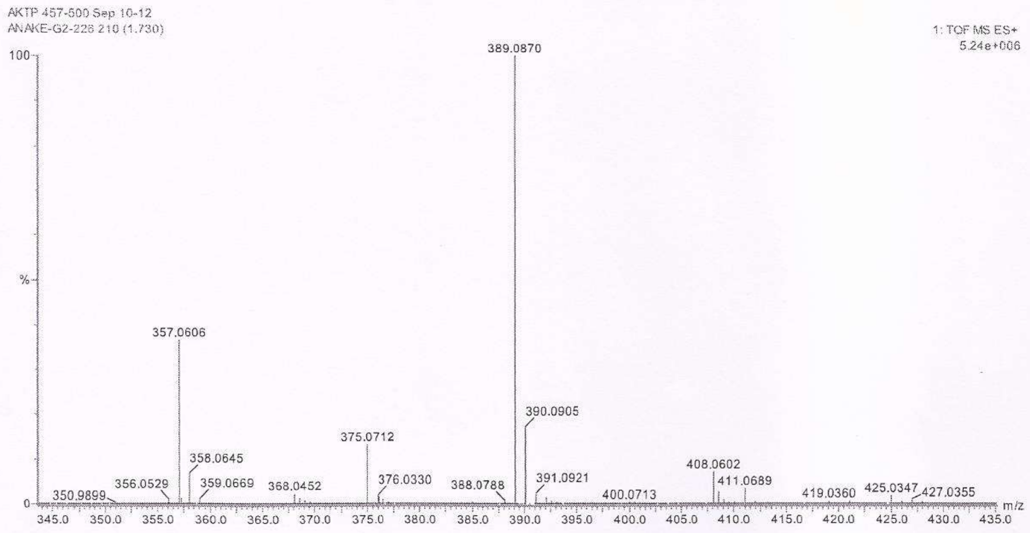

Figure S45. (+)-HRESIMS of **7.**

Elemental Composition Report [MH]+

Single Mass Analysis
Tolerance = 5.0 PPM / DBE: min = -1.5, max = 100.0
Element prediction: Off
Number of isotope peaks used for i-FIT = 3
Monoisotopic Mass, Even Electron Ions
27 formula(e) evaluated with 1 results within limits (up to 100 closest results for each mass)
Elements Used:
C: 28-28 H: 0-150 O: 0-30

Minimum:				-1.5				
Maximum:		5.0	5.0	100.0				
Mass	Calc. Mass	mDa	PPM	DBE	i-FIT	Norm	Conf(%)	Formula
425.3053	425.3056	-0.3	-0.7	8.5	810.3	n/a	n/a	C28 H41 O3

Elemental Composition Report [MNa]+

Single Mass Analysis
Tolerance = 5.0 PPM / DBE: min = -1.5, max = 100.0
Element prediction: Off
Number of isotope peaks used for i-FIT = 3
Monoisotopic Mass, Even Electron Ions
55 formula(e) evaluated with 1 results within limits (up to 100 closest results for each mass)
Elements Used:
C: 28-28 H: 0-150 O: 0-30 Na: 0-1

Minimum:				-1.5				
Maximum:		5.0	5.0	100.0				
Mass	Calc. Mass	mDa	PPM	DBE	i-FIT	Norm	Conf(%)	Formula
447.2870	447.2875	-0.5	-1.1	8.5	576.6	n/a	n/a	C28 H40 O3 Na

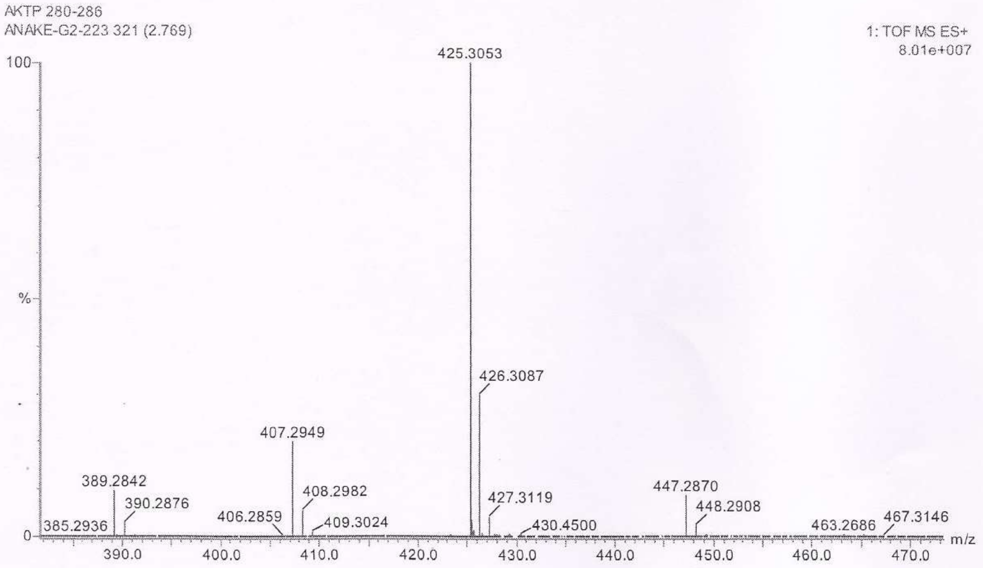

Table S1. ^{1}H and ^{13}C NMR data ($CDCl_3$, 300 and 75 MHz), COSY and HMBC for **AKTP 93-102P** (**8**).

Position	δ_C, type	δ_H, (*J* in Hz)	COSY	HMBC
1	164.9, CO	-		
3	148.2, C	-		
4	144.7, C	-		
4a	134.5, C	-		
5	103.1, CH	6.68, d (2.3)		C-4, 6, 7, 8a
6	166.8, C	-		
7	103.2, CH	6.69, d (2.3)		C-5, 6,8a,
8	165.3, C	-		
8a	100.8, C	-		
9	195.3, CO	-		
10a<br>b	42.8, CH_2	2.95, dd (19.0, 6.5)<br>2.31, dd (19.0, 1.4)	H-10b<br>H-10a	C-1, 4<br>C-1, 4
11	28.4, CH	3.44, dq (6.5, 1.4)	H-10a, 10b, 12	C-3, 4, 9
12	21.0, CH_3	1.46, d (6.9)	H-11	C-4, 10, 11
OMe-6	56.0, CH_3	3.94, s		C-6
OH-8	-	11.33, s		C-7, 8, 8a
